# Cellular and molecular synergy in AS01-adjuvanted vaccines results in an early IFNγ response promoting vaccine immunogenicity

**DOI:** 10.1038/s41541-017-0027-3

**Published:** 2017-09-08

**Authors:** Margherita Coccia, Catherine Collignon, Caroline Hervé, Aurélie Chalon, Iain Welsby, Sophie Detienne, Mary J. van Helden, Sheetij Dutta, Christopher J. Genito, Norman C. Waters, Katrijn Van Deun, Age K. Smilde, Robert A. van den Berg, David Franco, Patricia Bourguignon, Sandra Morel, Nathalie Garçon, Bart N. Lambrecht, Stanislas Goriely, Robbert van der Most, Arnaud M. Didierlaurent

**Affiliations:** 1 GSK Vaccines, Rixensart, Belgium; 2 Institute for Medical Immunology, Université libre de Bruxelles, Gosselies, Belgium; 3 Vlaams Instituut voor Biotechnologie, Inflammation Research Center, Ghent University, Ghent, Belgium; 4 Malaria Vaccine Branch, Military Malaria Research Program, Walter Reed Army Institute of Research, Silver Spring, Maryland USA; 5 Department of Methodology and Statistics, Tilburg University, Tilburg, The Netherlands; 6 Department of Psychology, Katholieke Universiteit Leuven, Leuven, Belgium; 7 Biosystems data analysis, Swammerdam Institute for Life Sciences, University of Amsterdam, Amsterdam, The Netherlands

## Abstract

Combining immunostimulants in adjuvants can improve the quality of the immune response to vaccines. Here, we report a unique mechanism of molecular and cellular synergy between a TLR4 ligand, 3-*O*-desacyl-4’-monophosphoryl lipid A (MPL), and a saponin, QS-21, the constituents of the Adjuvant System AS01. AS01 is part of the malaria and herpes zoster vaccine candidates that have demonstrated efficacy in phase III studies. Hours after injection of AS01-adjuvanted vaccine, resident cells, such as NK cells and CD8^+^ T cells, release IFNγ in the lymph node draining the injection site. This effect results from MPL and QS-21 synergy and is controlled by macrophages, IL-12 and IL-18. Depletion strategies showed that this early IFNγ production was essential for the activation of dendritic cells and the development of Th1 immunity by AS01-adjuvanted vaccine. A similar activation was observed in the lymph node of AS01-injected macaques as well as in the blood of individuals receiving the malaria RTS,S vaccine. This mechanism, previously described for infections, illustrates how adjuvants trigger naturally occurring pathways to improve the efficacy of vaccines.

## Adjuvants: Vaccine components working in synergy to improve beneficial effects of vaccination

A mechanism is revealed by which vaccine components co-operate to stimulate the immune system and improve vaccine efficacy. Some vaccines are formulated with adjuvants—compounds that induce a greater immune response to the vaccine and help to elicit greater protection against future infections. Arnaud Didierlaurent and his team of researchers at GSK Vaccines, Belgium, demonstrate that the two immunostimulants in the adjuvant AS01, used in several recently developed vaccines, works in tandem to trigger the activation of important immune system moderators. The synergistic effect of the immunostimulants modulate specific immune cells at the site of the vaccination to better prepare the body against future infection. Studies such as this allow us to better understand how vaccines work and lay the foundation for more informed research into future vaccine development.

## Introduction

Adjuvants are included in vaccines to improve humoral and cellular immune responses, particularly with poorly immunogenic subunit vaccines. Similar to natural infections by pathogens, adjuvants activate the innate immune system to promote long-lasting adaptive immunity.^1^ As simultaneous activation of multiple innate pathways is a feature of infections, adjuvants such as the Adjuvant Systems combine multiple immunostimulants to promote adaptive immune responses to vaccination.^1^

The Adjuvant System AS01 is a liposome-based adjuvant which contains two immunostimulants, 3-*O*-desacyl-4’-monophosphoryl lipid A (MPL) and QS-21.^2^ MPL is a non-toxic derivative of the lipopolysaccharide from *Salmonella minnesota*. QS-21 is a saponin fraction extracted from *Quillaja saponaria* Molina.^2^ AS01 is included in the recently developed malaria vaccine RTS,S (*Mosquirix*)^3^ and in other candidate vaccines in development against herpes zoster *(*HZ/su),^4^ HIV,^5^ and tuberculosis.^6^ The promotion of antigen-specific CD4^+^ T cells in addition to antigen-specific antibodies sets AS01 apart from other adjuvants.^2^ AS01-adjuvanted vaccines have also been effective in challenging populations, such as infants (with RTS,S against malaria^3^) and the older adults (HZ/su against herpes zoster^4^).

AS01 injection results in rapid and transient activation of innate immunity in animal models. Activated MHCII^high^ dendritic cells (DCs), which are necessary for T-cell priming, neutrophils and monocytes are rapidly recruited to the draining lymph node (dLN).^7^ It is also recognized that the two components of AS01 can have distinct functions. MPL signals via TLR4, stimulating NF-ĸB transcriptional activity and cytokine production and directly activates antigen-presenting cells (APCs) both in human and in mice.^8^ QS-21 can activate the inflammasome in vitro^9^ and promotes high antigen-specific antibody and CD8^+^ T-cell responses in mice and antigen-specific antibody responses in humans.^10^ However, what is less clear is how immunostimulants function in combination.

Here, we investigate how combining immunostimulants results in complex patterns of innate immune activation. We present evidence from mouse, non-human primate and clinical studies that the MPL-QS-21 combination in AS01 results in the stimulation of de novo pathways that were not triggered by the individual components, but were essential for the promotion of a polyfunctional CD4^+^ T-cell response and Th1-antibody isotype switching. We also reveal that AS01 triggers naturally occurring defense mechanisms, including NK-cell and innate-like CD8^+^ T-cell activation in the lymph node at the earliest stages of the innate immune response. Finally, we have shown that those mechanisms play a role in protection conferred by AS01-containing vaccine.

## Results

### The combination of MPL and QS-21 induces potent adaptive immune response to AS01-adjuvanted vaccines

**Fig. 1:**
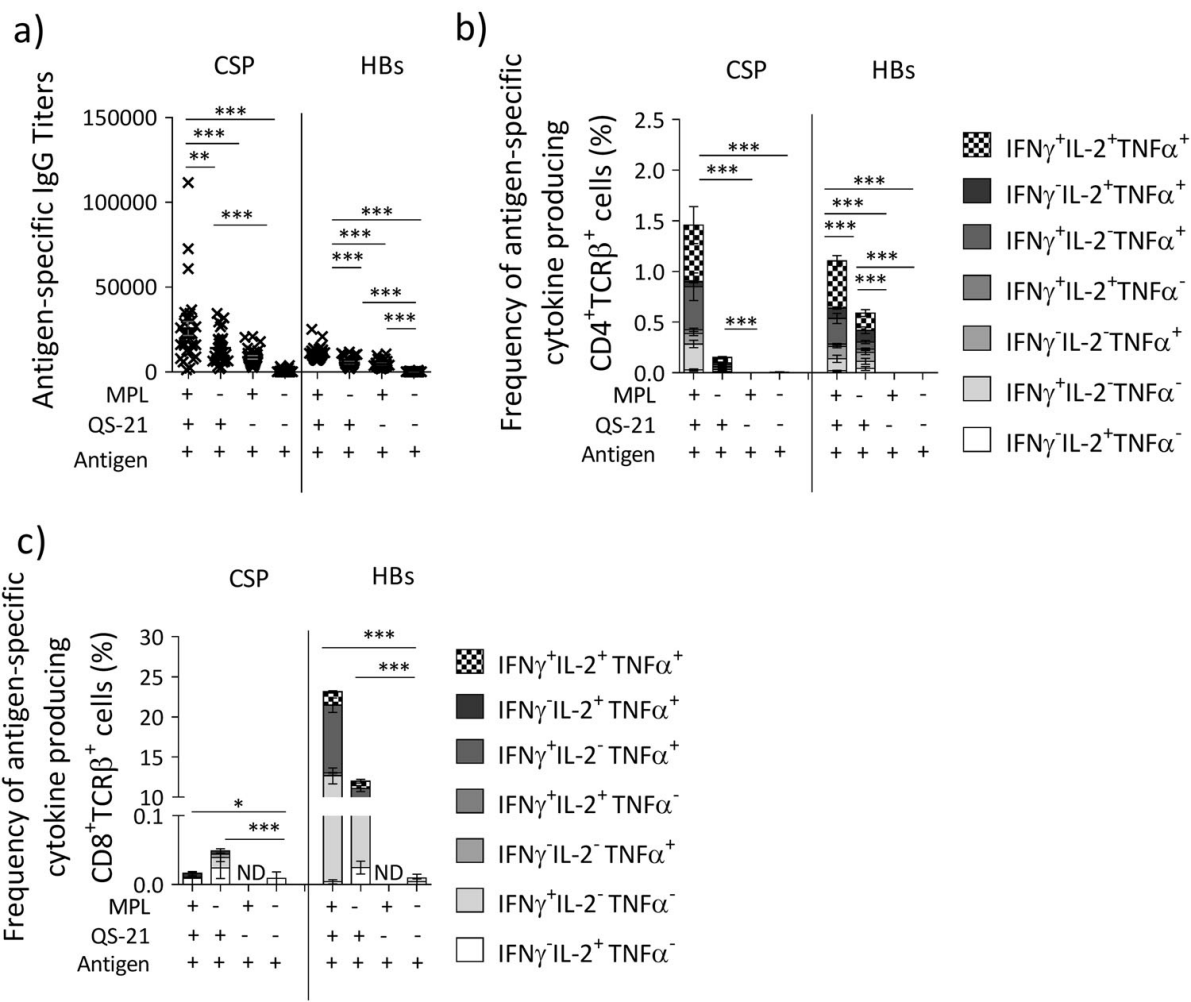
MPL and QS-21 act synergistically to induce strong antigen-specific CD4^**+**^ T-cells and antibody responses. C57BL/6 mice were immunized twice 2 weeks apart with the RTS,S antigen formulated together with indicated adjuvants (RTS,S = 50 µg/ml, AS01 = MPL + QS-21, MPL = 50 µg/ml, QS-21 = 50 µg/ml, 2 × 50 µl/injection, i.m.). **a** 14 days after the last immunization, antigen-specific IgG titers were assessed by ELISA (*n* = 30 mice/group, 1 out of 2 independent experiments is shown). **b**, **c** Spleens were obtained 7 days after the last dose of vaccine and splenocytes were restimulated with a pool of peptide spanning the length of HBs or CSP. **b** Antigen-specific cytokine production by CD4^+^ T cells as assessed by ICS. **c** Antigen-specific cytokine production by CD8^+^ T cells as assessed by ICS (*n* = 5 mice/group, 1 out of 2 independent experiments is shown). *Bar charts* show mean ± SEM

We initially assessed the contribution of MPL and QS-21 in the adjuvant effect of AS01 using the RTS,S vaccine. The RTS,S antigen consists of a fusion of the central repeat and C-terminal flanking regions of *Plasmodium falciparum* circumsporozoite protein (CSP) with the hepatitis B surface antigen (HBs), coexpressed with HBs alone. Naive wild-type (WT) mice were immunized with RTS,S formulated either without adjuvant, with MPL, with QS-21 (both formulated in liposomes) or with AS01. Two immunizations were administered 2 weeks apart and the HBs- and CSP-specific immune response (serum IgG and T-cell responses) was measured after the second dose. RTS,S/AS01-induced antigen-specific IgG responses were higher than those induced by either RTS,S/MPL or RTS,S/QS-21 (Fig. 1a). RTS,S/AS01 also induced higher antigen-specific CD4^+^ and CD8^+^ T-cell responses than RTS,S/QS-21, whereas RTS,S/MPL induced virtually no antigen-specific T-cells (Fig. 1b, c). Notably, very low levels of CD8^+^ T-cell responses were generated against CSP (Fig. 1c), as previously reported.^11^ Additionally, RTS,S/AS01 induced predominantly a Th1 response,^12^ with a relatively high prevalence of IFNγ^+^IL-2^+^TNFα^+^ phenotype (Fig. 1b), as reported for other antigens.^12, 13^ As previous reports suggested that triple-positive CD4^+^ T cells produce higher levels of cytokines,^14^ we assessed this in WT mice immunized with HBs/AS01 on day 0 and day 14 (Supplementary Fig. 1). HBs is the antigen used in the *Engerix* vaccine and it induced cellular responses in mice vaccinated with RTS,S/AS01(Fig. 1b, c). For each CD4^+^ T-cell phenotype, the mean fluorescence intensity (MFI) of each cytokine was measured as a proxy for the level of protein production. This analysis confirmed that the triple-positive IFNγ^+^IL-2^+^TNFα^+^ CD4^+^ T cells produced the highest levels of each of the cytokines, in comparison with single- or double-positive T cells (Supplementary Fig. 1). Hence, combining MPL and QS-21 in AS01 results in enhanced adaptive immune responses to RTS,S in comparison with the individual immunostimulants.

### Complex interplay between MPL and QS-21 in AS01 transcriptional response

**Fig. 2:**
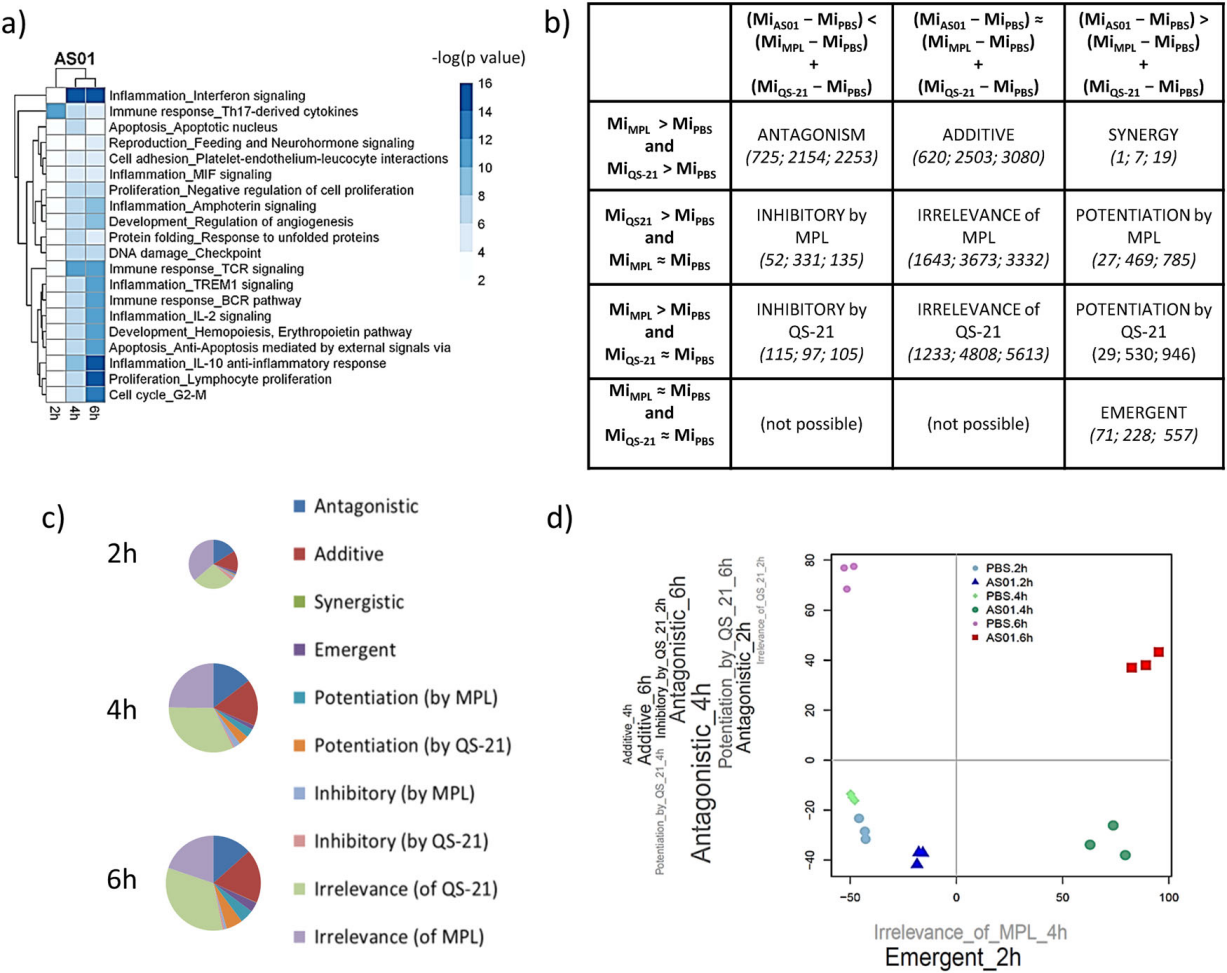
Statistical analysis reveals multiple types of interplays between the components of AS01. C57BL/6 mice (*n* = 3/adjuvant/time point) were immunized once with HBs + AS01 = MPL + QS-21, MPL = 100 µg/ml, QS-21 = 100 µg/ml, 2 × 50 µl injections, i.m). Draining lymph nodes (dLNs) were isolated at indicated time points and gene expression was assessed by microarray analysis. Linear models and contrast analysis were used to identify differentially expressed genes (DEGs, cutoffs: fold Change > 2, *p*-value for contrast  < 0.01, adjusted for multiple comparisons). **a** Functional analysis of DEGs was performed in MetaCore. The top 10 most enriched process networks for each time point are presented. Hierarchical clustering by column was performed to highlight similarities between time points. **b** A taxonomy of possible interplays between QS-21 and MPL in AS01 is shown. The conditions to be met for each category are represented in bold. *M*_i_ represents the gene expression level for each gene i. Numbers in italics represent the number of AS01 DEGs belonging to each category at 2 h, 4 h, and 6 h after immunization, respectively. **c** Plots represent the distribution of AS01 DEGs among the different interplay catagories. The size of each pie is proportional to the total number of DEGs at that time point. **d** Principal componant (PC) analysis was used to reduce the dimensionality of the DEG list. PC1 and PC2 are represented here, together with the scores of each sample. *Word Clouds* represent the interplay enriched in each PC’s loadings. The size of the character is proportional to the –log(*p-*value) of the enrichment, while the intensity of the color is proportional to the effect size

To investigate the molecular pathways induced by AS01, we analyzed the gene expression profile in the dLN of C57BL/6 mice injected i.m. with AS01 after 2, 4, and 6 h. Bioinformatics analysis identified a number of genes differentially expressed over sham treatment (differentially expressed genes = DEG; log_2_(fold change) > 2; *p* < 0.01; Supplementary Figure 2A). Pathway enrichment analysis of DEG showed that the response to AS01 was concentrated at 4 and 6 h, with few pathways enriched at 2 h (Fig. 2a). A notable exception was the pathway “*Immune response – Th17 related cytokines*”, which includes multiple pleiotropic innate cytokines (Supplementary Fig. 2B), confirming that AS01 rapidly activates innate immunity in the dLN.^7^ The processes enriched by AS01 included those related to cytokines, such as interferons, MIF and IL-2. In particular, the interferon-signaling pathway was the most highly enriched pathway at 4 and 6 h, and contributed the most to the variability in the microarray data set (Supplementary Fig. 2C). Moreover, at 6 h, an IL-10-driven anti-inflammatory pathway was also induced by AS01.

To understand the contribution of MPL and QS-21 to gene expression induced by AS01, we compared the transcription response of the three adjuvants (pathways enriched in MPL and QS-21-induced responses are described in Supplementary Fig. 2D). To go beyond the simple pairwise comparison between immunostimulants, we used a statistical approach initially designed for the analysis of combination drugs.^15^ This approach categorizes the potential contributive relationships that MPL and QS-21 provide in AS01 for each gene expressed (Fig. 2b, examples are shown in Supplementary Fig. 2D). As shown by the high proportion of genes falling into the *irrelevance of MPL* or *irrelevance of QS-21* categories, over half the genes affected by AS01 can be ascribed as MPL-specific or QS-21-specific (Fig. 2c). Altogether, those MPL- or QS-21-specific genes were generally more frequent than genes affected in an additive fashion by MPL and QS-21. Beyond the simple additive effect, a number of genes were affected in a synergistic fashion and interestingly, we also identified a group of genes which were not induced by either QS-21 or MPL immunization alone, but which were present in AS01-immunized animals (Fig. 2c). We classified these genes as “emergent”.

Principal component analysis (PCA) was then used to assess which relationships contributed the most to the variation in gene expression over time after injection of AS01 or PBS. The first 2 principal components (PCs) accounted for 78% of the variance (PC1 = 58%, PC2 = 20%; see bivariate plot Fig. 2d). Projecting each sample in this plot showed good separation between PBS and AS01 samples (across PC1), and between samples at different time points (across PC2). Further analysis showed that PC1 was enriched in early emergent genes, together with genes driven by QS-21 (as shown by enrichment in the *irrelevance of MPL* category). PC2 data were instead enriched with genes driven by antagonistic and additive interplays, together with QS-21-driven interplays such as *potentiation by QS-21* and *inhibitory by QS-21*. Overall, this analysis highlights the importance of emergent interplays and QS-21-driven effects in the transcriptional response to AS01.

### Innate IFNγ promotes adaptive immune response to AS01 adjuvanted antigens

**Fig. 3:**
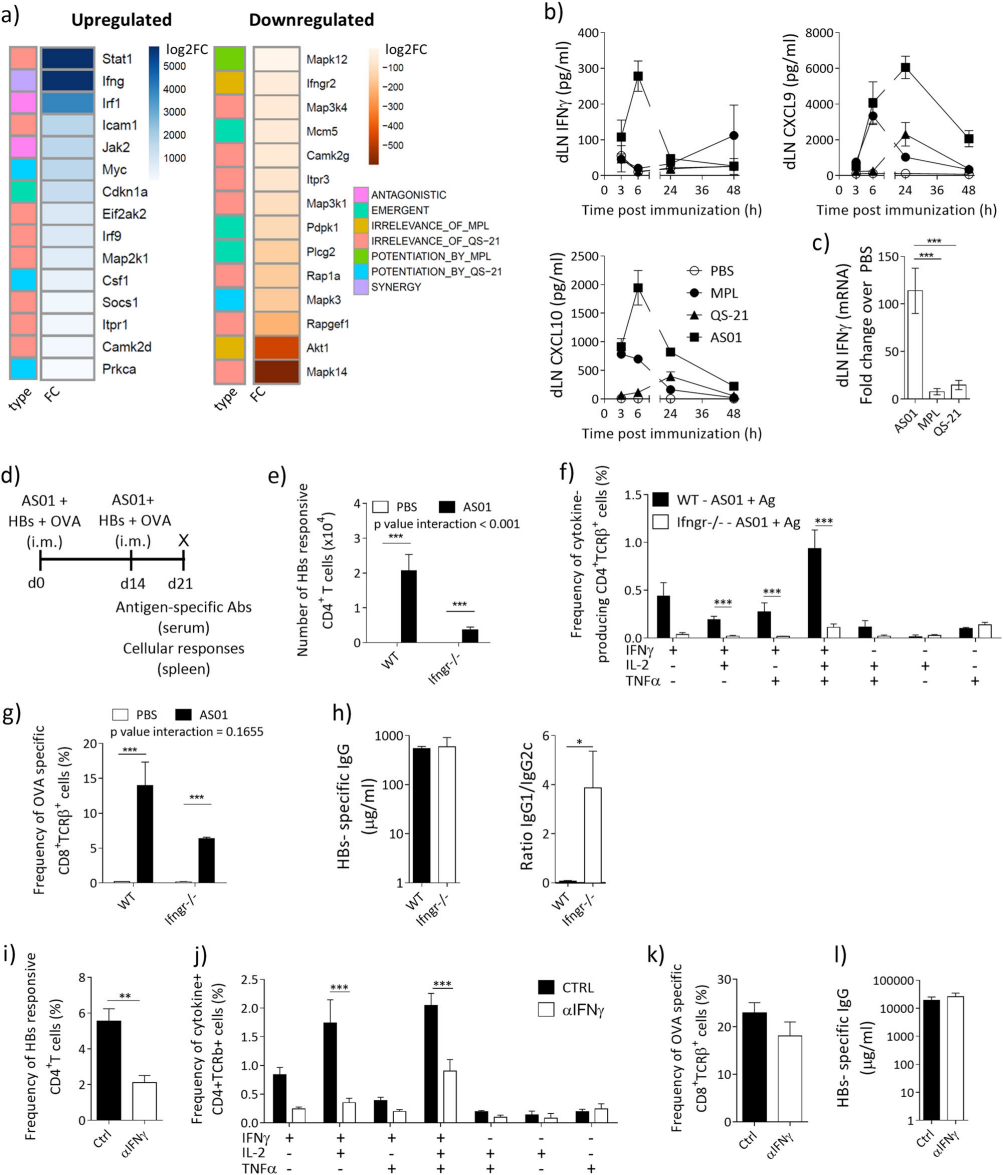
IFNγ is required for the induction of adaptive immune responses after immunization with an AS01-adjuvanted vaccine. **a** Transcriptional analysis was performed as in Fig. 2. DEGs at 4 h post immunization in the “Inflammation interferon_signalling” process network (MetaCore) are represented in a heatmaps showing their −log2FC over PBS and their interplay classification. **b**, **c** C57BL/6 mice were immunized once with HBs + indicated adjuvants (AS01 = MPL + QS-21 MPL = 100 µg/ml, QS-21 = 100 µg/ml, 2 × 10 µl injections, i.m.). dLNs were isolated at indicated time points and homogenized. **b** Cytokine concentration as assessed by Luminex (*n* = 5 mice/group, 1 out of 2 independent experiments is shown). **c** Levels of IFNγ mRNA transcript in the dLN as measured by RT-PCR (*n* = 5 mice/group). **d** Timeline of immunization protocol. Mice were immunized with AS01 and HBs/OVA (HBs = 80 µg/ml; OVA = 20 µg/ml; AS01 = 100 µg/ml MPL + 100 µg/ml QS-21, 2 × 50 µl/injection i.m.) twice at a 2 week interval. At day 21 levels of antigen-specific antibodies were titrated in the serum. Spleens were collected and splenocytes were restimulated with a pool of peptide spanning the length of HBs or OVA and analyzed by ICS. **e**–**g** C57BL/6 or *Ifngr*^*−/−*^ were immunized as in **c**. *N* = 6/condition, one out of two independent experiment is shown. **e** Frequency of total cytokine-producing antigen-specific CD4^+^ T cell. **f** Frequency of antigen-specific CD4^+^ cytokine-producing species. **g** Frequency of OVA-specific SIINFEKL-H2Kb^+^ CD8^+^ T cells. **h** Levels of HBs-specific IgG and ratio of HBs-specific IgG1/IgG2c in the serum. **i**–**k** WT mice were treated with an αIFNγ mAb (XMG1.2, 100 µg/mouse i.p.) and 48 h later immunized with AS01 and HBs/OVA as in **d**. **i** Frequency of total cytokine-producing antigen-specific CD4^+^ T cells. **j** Frequency of antigen-specific CD4^+^ T cells by cytokine expression. **k** Frequency of OVA-specific SIINFEKL-H2Kb^+^ CD8^+^ T cells. **l** HBs-specific IgG measured by ELISA in the serum. (*N* = 5/condition, one representative out of two independent experiments is shown. All graphs show mean ± SEM

Our analysis showed that interferon-related signals were enriched in the transcriptional response to AS01 (Fig. 2a and Supplementary Fig. 2C). A more focused evaluation of this signature at 4 h revealed a strong, synergistic induction of *Ifng* and the induction of number of genes in its signaling pathway, including *Stat1*, *Jak2*, and *Ifngr2* (Fig. 3a). Noticeably, genes in this pathway fell into a number of interplay categories, highlighting how within a same function, MPL and QS-21-mediated signals contribute in different ways to the response to AS01.

Numerous studies indicate an important role for innate immune-derived IFNγ in the induction of protective CD4^+^ T-cell responses to infections.^16–19^ We have previously shown that the innate immune response to AS01, but not to AS04 or emulsion-based adjuvants, is characterized by IFNγ-related cytokine production in the dLN.^7, 20, 21^ To confirm the synergistic induction of IFNγ by MPL and QS-21 in AS01, we analyzed the levels of this cytokine in the dLN after immunization with HBs adjuvanted with AS01 or its components. AS01-induced the early production of IFNγ and the IFNγ-related cytokines CXCL9 and CXCL10, with IFNγ and CXCL10 concentrations peaking at 6 h (Fig. 3b). In comparison, MPL and QS-21 also induced CXCL9 and CXCL10 production, albeit at concentrations lower than those observed with AS01. However, they failed to significantly induce IFNγ (Fig. 3b). AS01 also induced significantly higher level of IFNγ mRNA at 4 h than MPL or QS-21 alone (Fig. 3c).

The requirement for IFNγ in the induction of an adaptive immune response was first assessed in IFNγR-deficient mice. Mice received two i.m. injections on Day 0 and Day 14 of AS01-adjuvanted or unadjuvanted vaccines containing two antigens, HBs and ovalbumin (OVA). HBs was selected because it is clinically relevant, and OVA was selected because it allows Ag-specific CD8^+^ T cells to be detected using SIINFEKL-H2Kb pentamers. On Day 21, splenocytes were restimulated with HBs peptides and analyzed by intracellular cytokine staining (ICS) (Fig. 3d). As expected, the AS01-adjuvanted vaccine (HBs + OVA/AS01) elicited much stronger HBs-specific CD4^+^ and CD8^+^ T-cell responses than the non-adjuvanted vaccine (Fig. 3e–g). Immunization with HBs + OVA/AS01 induced very limited Th2 and Th17 responses, which were unaffected by IFNγR deficiency (Supplementary Fig. 3). In IFNγR-deficient mice, HBs + OVA/AS01 elicited a much lower HBs-specific CD4^+^ T-cell response than in WT mice, with CD4^+^ T cells producing more than one cytokine particularly affected (Fig. 3e, f). HBs + OVA/AS01 also elicited a lower (although not significant) OVA-specific CD8^+^ T-cell response than WT mice (interaction *p-*value = 0.16; Fig. 3g). The overall IgG titers to HBs + OVA/AS01 were unaffected by the IFNγR deficiency, but IFNγR^−/^^−^ mice showed decreased titers of Ag-specific IgG2c antibodies (Fig. 3h).

As the IFNγR deficiency may affect the response to vaccination beyond the activity of AS01 on the innate phase of the response, we depleted early IFNγ in the dLN by administrating a low dose of αIFNγ antibody (XMG1.2, 100 μg i.p.^22^) or an isotype antibody control to WT mice 2 days before vaccination with HBs + OVA/AS01. As shown in Fig. 3i, j, a single administration of αIFNγ mAb resulted in lower HBs-specific CD4^+^ T-cell responses in IFNγ-depleted mice than in control mice. By contrast and similar to what was observed in the IFNγR-deficient mice, the αIFNγ mAb did not significantly lower OVA-specific CD8^+^ T-cell responses or humoral responses (Fig. 3k, l). Overall, these data indicate that IFNγ production in the early phase after vaccination is important for the induction of polyfunctional CD4^+^ T-cell response (mainly of a Th1 phenotype) and impact the quality of the antibody response to AS01-adjuvanted antigens.

**Fig. 4:**
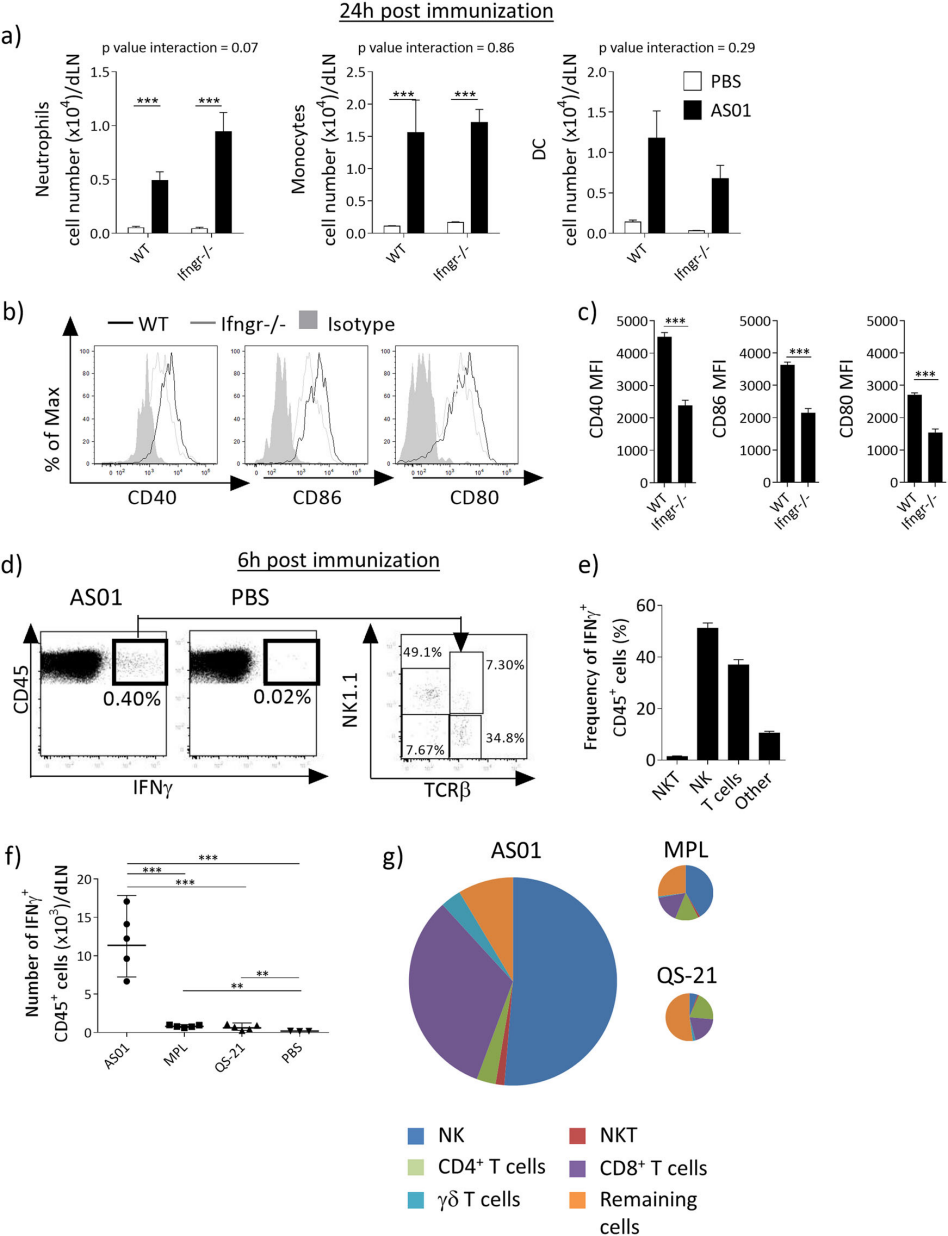
IFNγ is required for the recruitment and activation of APCs in the dLN after immunization with an AS01-adjuvanted vaccine. WT or Ifngr^−/−^ mice were immunized with AS01 + HBs (HBs = 100 µg/ml, 2 × 50 µl/injection i.m.). Twenty-four hours after immunization, dLNs were collected and innate immune populations were evaluated by FACS. **a** Numbers of neutrophils (CD45^+^CD11b^+^Ly6G^Hi^), monocytes (CD45^+^CD11b^+^Ly6C^Hi^) and DCs (CD45^+^CD11c^+^MHCII^Hi^) (*N* = 4 for PBS injected and 5 for AS01 injected, 1 representative out of 2 independent experiments is shown). **b**, **c** Mean Fluorescence Intensity (MFI) for indicated maturation markers on DCs. **b** Representative FACS histograms. **c** Quantification. (*N* = 5, 1 representative plot is shown). **d**–**g** C57BL/6 mice were immunized with AS01 + HBs as above. Six hours later, dLNs were collected and the frequency and identity of IFNγ producing cells was assessed by ICS. **d** Representative FACS plots of all live cells showing IFNγ^+^ CD45^+^ cells and their identity. One representative out of four independent experiments is shown. **e** Distribution of IFNγ^+^ cells among different cell population. **f** C57BL/6 mice were immunized with AS01 or its components (AS01 = MPL + QS-21 MPL = 100 μg/ml, QS-21 = 100 μg/ml, 2 × 10 μl injections, i.m.). After 6 h, dLNs were collected and IFNγ^+^ production assessed by ICS. **g** Pie charts showing the distribution of IFNγ^+^ cells among different cell types depending on immunostimulant used. The area of the chart is proportional to the number of IFNγ^+^ cells for each condition (1 out of 2 independent experiments is shown). *Bar graphs* show mean ± SEM. Data were analyzed by one-way or two-way ANOVA followed by Bonferroni post hoc test to compare all groups or by unpaired Student's *t*-test, as appropriate

Given that IFNγ is known to modulate the function of APCs, we addressed its role in promoting the accumulation and maturation of innate immune cells in the dLN after injection of AS01. We have previously shown that AS01 induces the recruitment of neutrophils, monocytes, and DCs in the dLN, peaking at 24 h after injection.^7^ As expected, in the dLN of WT mice, HBs/AS01 elicited the recruitment of CD11b^+^ Ly6G^Hi^ neutrophils, CD11b^+^ Ly6C^Hi^ monocytes and CD11c^+^MHCII^Hi^ DCs compared with non-adjuvanted HBs (Fig. 4a). In IFNγR-deficient mice, HBs/AS01 elicited a similar recruitment of neutrophils and monocytes to that in WT mice, but elicited a lower (although not significant) recruitment of DCs (Fig. 4a). Additionally, these recruited DCs expressed lower levels of the costimulatory molecules CD40, CD86, and CD80 (Fig. 4b, c) than those DCs in WT mice. Overall, these data show that the early IFNγ signal in response to AS01 is important in promoting APC activation, which may explain the impact on T-cell and antibody responses described above.

### NK cells and CD8^+^ T cells produce IFNγ in the dLN within the first 6 h of the response to AS01

The source of early IFNγ production in the dLN after injection of AS01 was investigated by flow cytometry. This showed that CD45^+^ cells produce IFNγ after 6 h (Fig. 4d). The CD45^+^ IFNγ^+^ population was composed on average of ≈51% NK cells, ≈37% TCR αβT cells (mainly CD8^+^ T cells), ≈1.5% NKT cells and ≈10% of other cells (Fig. 4e). Overall, ≈33% of NK cells produced IFNγ, whereas only 0.2% of CD8^+^ T cells produced IFNγ. When compared with AS01, MPL or QS-21 induced only a minimal accumulation of IFNγ^+^ cells in the dLN at 6 h (Fig. 4f), confirming the synergistic induction of IFNγ by AS01. Moreover, the relative proportion of cells producing IFNγ differed in mice injected with AS01, MPL, or QS-21 (Fig. 4g). Notably, in mice injected with MPL, NK cells contributed to IFNγ production (≈41% IFNγ^+^CD45^+^ cells), whereas, in QS-21-injected mice, NK cells contributed little to IFNγ^+^ cells (≈6% IFNγ^+^CD45^+^ cells). The proportion of IFNγ^+^ CD8^+^ T cells differed between conditions (≈32% of IFNγ^+^CD45^+^ cells with AS01, ≈16% with MPL and ≈20% with QS-21); and the proportions of IFNγ^+^ NKT cells and IFNγ^+^ TCRγδ^+^ cells were generally low (NKT + TCRγδ^+^cells combined: ≈5% of IFNγ^+^CD45^+^ cells with AS01; ≈2% with MPL and ≈3% with QS-21). These data confirm that QS-21 and MPL in AS01 synergistically induce early IFNγ production by NK cells and CD8^+^ T cells in the dLN.

**Fig. 5:**
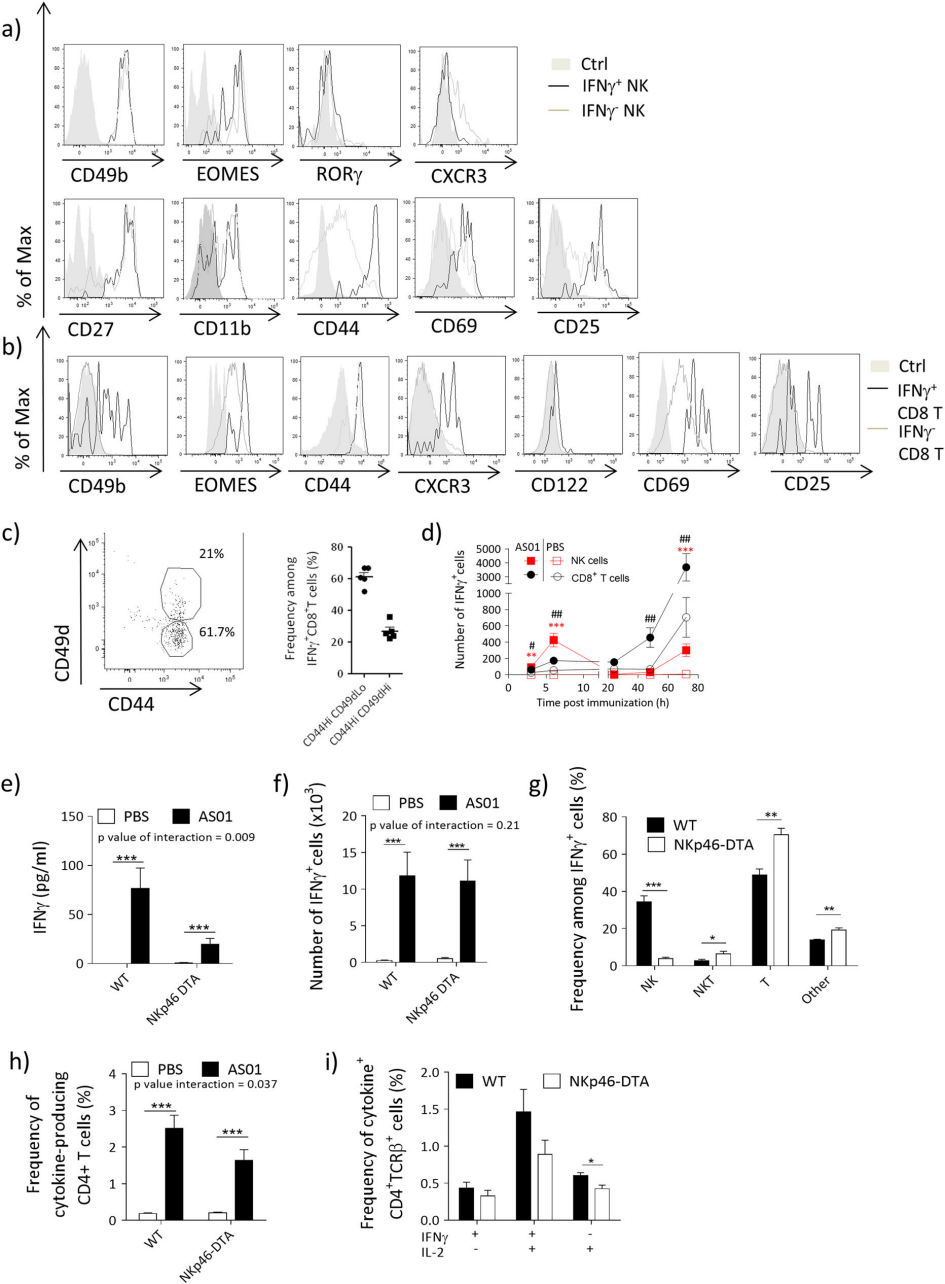
Characterization of IFNγ^**+**^ cells in the dLN. **a**–**c** C57BL/6 mice were immunized with AS01 + HBs (HBs = 100 μg/ml, 2 × 50 μl/injection i.m.). Six hours later, dLN were collected and the characteristics of IFNγ-producing cells were assessed by FACS. **a** Phenotype of IFNγ^+^ NK cells. **b**, **c** Phenotype of IFNγ^+^ CD8^+^ T cells. **d** C57BL/6 mice were treated as in **a** and the accumulation of IFNγ^+^ NK and IFNγ^+^ CD8^+^ T cells in the dLN at indicated time points was assessed (*n* = 4–8 from 1 of 2 independent experiments). Level of significance is indicated by * for NK cells and # for CD8^+^ T cells. **e**–**g** *NKp46(iCre)-ROSA-stop*^*flox-*^*DTA* (NKp46-DTA) or WT mice were immunized with AS01 + HBs/OVA (HBs 80 μg/ml, OVA 20 μg/ml, 2 × 25 μl/injection i.m.) and euthanized 6 h after immunization. **e** IFNγ levels in the serum. **f-g** dLN cell suspension was restimulated ex vivo and analyzed by ICS. **f** Number of IFNγ-producing cells in the dLN. **g** Contribution of different cell types to IFNγ^+^ cells. (*N* = 5/group). **h**–**i** NKp46-DTA or WT mice were immunized as in Fig. 3d. **h** Frequency of total cytokine-producing antigen-specific CD4^+^ T cells. **i** Frequency of antigen-specific CD4^+^ T cells by cytokine expression (*N* = 9/group). *Bar charts* show mean ± SEM. Data were analyzed by one-way or two-way ANOVA followed by Bonferroni post hoc test to compare all groups or by unpaired Student's *t*-test, as appropriate

We found that AS01-induced IFNγ^+^NK cells were CD49b^+^(DX5^+^), Eomes^+^, RORγ^-^, a phenotype compatible with conventional NK cells (Fig. 5a). Murine NK-cells developmental stages can be classified via the differential expression of the markers CD27 and CD11b.^23^ AS01-induced IFNγ^+^NK cells expressed high levels of CD27, and included both CD11b^+^ and CD11b^−^ populations, phenotypes consistent with a high cytokine-producing capacity (Fig. 5a).^23^ In addition, the high-expression levels of CD44, CD69, and CD25 in IFNγ^+^NK cells in comparison with IFNγ^-^ NK cells suggest that they are activated effector cells (Fig. 5a). By contrast, IFNγ^+^ NK cells expressed lower levels of the chemokine receptor CXCR3 than their IFNγ^-^ counterparts (Fig. 5a). IFNγ^+^ CD8^+^ T cells were CD49b^low^, Eomes^+^, CD122^+^, CXCR3^+^, and for the majority CD44^Hi^CD49d^low^ (Fig. 5b, c). Together with their rapid IFNγ response to AS01, this phenotype suggests that most CD8^+^ T cells are unconventional virtual memory T cells. As with IFNγ^+^ NK cells, IFNγ^+^ CD8^+^ T cells also expressed the activation markers CD25 and CD69 (Fig. 5b).

In the dLN and after the injection of AS01, the changes in the IFNγ^+^ subpopulation of NK cells differed from that of the entire NK cell population. The size of IFNγ^+^ NK-cell subpopulation was largest at 6 h, returning to baseline at 24 h, while the number of total NK cells remained the same within the same timeframe (Supplementary Fig. 4A). Both populations then steadily increased by 48 h. By contrast, the size of IFNγ^+^ CD8^+^ T-cell subpopulation steadily increased from 3 h and paralleled that of the entire CD8^+^ T-cell population (Fig. 5d). Finally, the induction of IFNγ^+^ cells was unaffected across a range of AS01 doses (dilutions up to 25-fold of AS01, Supplementary Fig. 4B), excluding the possibility that the synergistic aspect of the induction of IFNγ by AS01 was dose dependent.

As NK cells were the major IFNγ-producing cells in the dLN, we assessed the effect of NK-cell deficiency on the response to AS01-adjuvanted vaccines. For this purpose, we used a cre-lox mouse model, in which the lethal diptheria toxin subunit was expressed under the control of the NK-cell-specific Nkp46 promotor (NKp46-DTA mice).^24, 25^ As expected, NKp46-DTA mice lacked NK cells in the dLN (Supplementary Fig. 4C). After immunization with HBs/AS01, serum IFNγ levels were lower in the NK-cell-deficient mice than in WT mice (Fig. 5e). In the dLN, although IFNγ production by NK cells was abrogated, the levels of IFNγ^+^ CD45^+^ cells were similar in the NK-cell-deficient mice to that in WT mice, mainly due to compensatory production by other cells, including T cells (Fig. 5f, g). After immunization with HBs + OVA/AS01 (as in Fig. 3d), NK-cell deficiency resulted in a mild but significant reduction in antigen-specific CD4^+^ T-cell responses in the NKp46-DTA mice in comparison with littermate controls (Fig. 5h, i), suggesting a partial role for NK cells in promoting CD4^+^ T-cell response to vaccination. Antigen-specific CD8^+^ T cells and antibody responses were not affected in this model (data not shown).

### Early IFNγ induction by AS01 is mediated by subcapsular macrophages via IL-18 secretion, and supported by IL-12

**Fig. 6:**
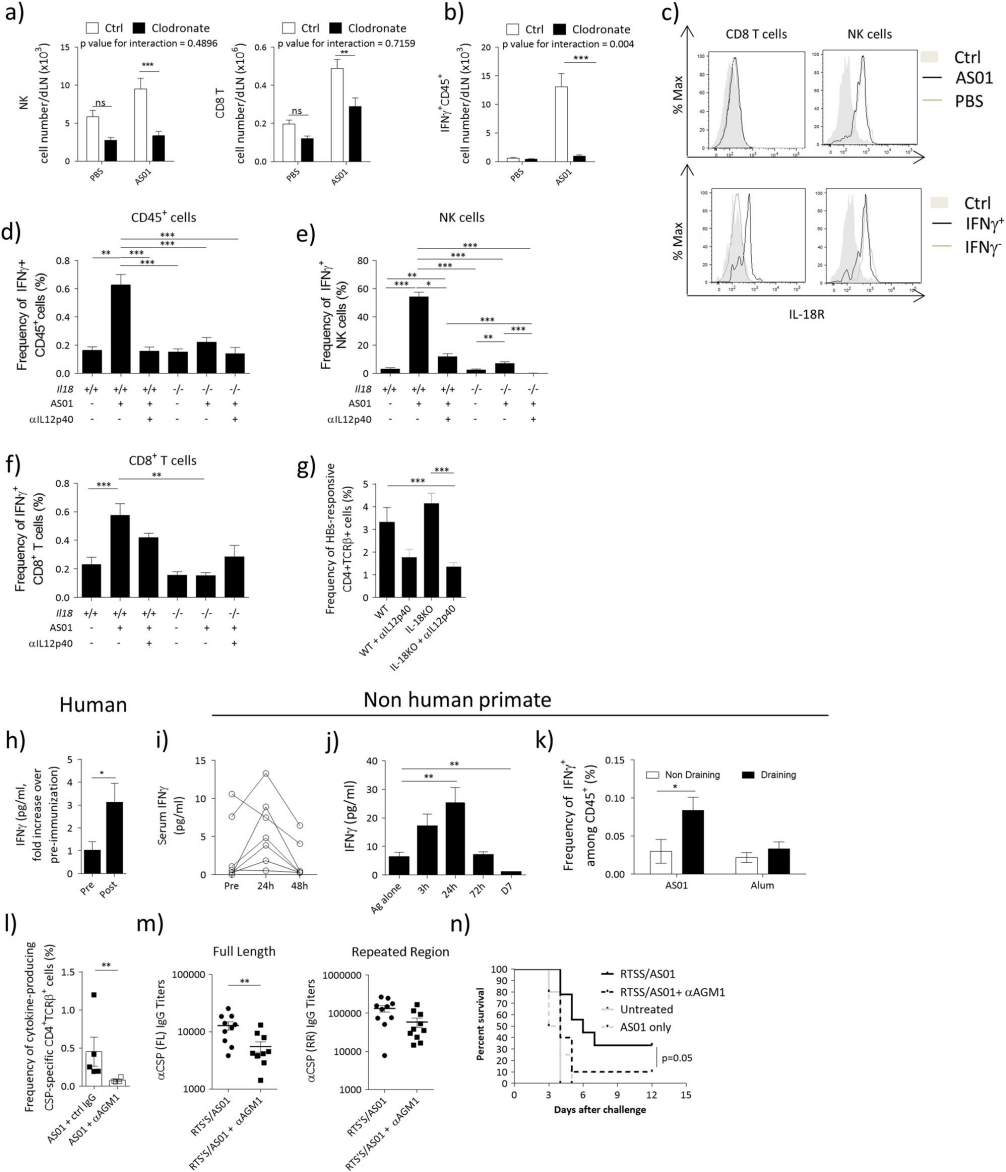
Innate IFNγ production is mediated by SSM-derived IL-18, in combination with IL-12. **a**, **b** C57BL/6 were treated with 20 μl of clodronate liposomes or control liposomes i.m. Six days later, mice were immunized with AS01 + HBs (HBs 100 μg/ml, 2 × 10 μl/injection i.m.). Six hours after immunization, dLNs were collected and analyzed by FACS. **a** Effect of subcapsular sinus macrophages depletion on NK (*left*) and CD8^+^ T cells (*right*). **b** Number of IFNγ^+^ CD45^+^ cells. **c** WT mice were immunized with AS01 + PBS or HBs (HBs 100 μg/ml, 2 × 50 μl/injection i.m.). dLNs were collected 6 h after immunization and IL-18R expression was assessed by FACS. *Top panels* show the expression of IL-18R on CD8^+^ T cells or NK cells after immunization. The *bottom panel* shows IL-18R expression on IFNγ^-^ or IFNγ^+^ populations. **d**–**f** C57BL/6 WT or *Il18*^*−/−*^ mice were treated with αIL-12p40 (C17.8, 0.5 mg, i.p.) and immunized the next day with AS01 + HBs. dLNs were collected 6 h after immunization. The accumulation and frequency of IFNγ**-**producing cells was evaluated by ICS. **d** Frequency of CD45^+^ IFNγ^+^ cells. **e** Frequency of IFNγ^+^ NK cells. **f** Frequency of IFNγ^+^CD8^+^ T cells. (*N* = 4–20, data from 2 pooled independent experiments are shown). **g** C57BL/6 WT or *Il18−/−* mice were treated with αIL-12p40 (C17.8, 0.5 mg, i.p.) and immunized the next day with AS01 + HBs/OVA (HBs 80 μg/ml, OVA 20 μg/ml, 2 × 50 μl/injection i.m.). Mice were immunized twice at 14 days intervals. On Day 21, spleens were collected and frequency of total cytokine-producing antigen-specific CD4^+^ T cells were anlysed by ICS (*n* = 8, 2 pooled experiments are shown). **h** Healthy individuals were immunized with RTS,S/AS01 (per 0.5 ml dose: 50 μg of RTS,S, AS01 = 50 μg of MPL and 50 μg of QS-21, i.m.). The concentration of IFNγ in the serum was evaluated by ELISA. Data show fold increase in the levels of IFNγ in the serum before immunization (pre) or 24 h after injection (*N* = 33). **i**–**k** Chinese rhesus macaques were immunized i.m. with AS01 (2 × 250 μl) + VZV glycoprotein E (25 μg) (**i**, **j**) or HIV recombinant protein F4 (10 μg) (**k**). Cytokine levels were analyzed by multiplex bead assay and ICS. **i** Levels of IFNγ in the serum at indicated time points. **j** Levels of IFNγ in the dLN at indicated time points. **k** Frequency of IFNγ-producing cells in draining and non-draining lymph node 24 h after immunization. For sera *N* = 7, for dLN *N* = 2–4, for ICS *N* = 3–6. **l**–**n** Mice were immunized with RTS,S/AS01 as in Figure 3D (1 × 50 μl, i.m.). Two days before immunization and on the day of immunization, mice were administered 100 μl of α-AGM1 Ab or control rabbit polyclonal serum. **l** On Day 21, spleens were collected and splenocytes were restimulated with a pool of peptide spanning the length of CSP and frequency of total cytokine-producing antigen-specific CD4^+^ T cells were analyzed by ICS. **m** IgG titers against full length or the NANP repeated reagions of CSP. **n**. Two weeks after the last immunization, mice were infected with 3000 transgenic *Plasmodium berghei* sporozoites expressing *Plasmodium falciparum* CSP. Mice were monitored daily and considered infected after showing parasitemia by blood smear for 2 consecutive days. Kaplan–Meyer survival curves are shown (*N* = 10/group). All *graphs* show mean ± SEM. Data were analyzed by one-way or two-way ANOVA followed by Bonferroni post hoc test, by Student's *t*-test or by paired Student's *t*-test, as appropriate. *p<0.05, **p < 0.01, ***p < 0.001. All *charts* show mean ± SEM

Subcapsular sinus macrophages (SSM) promote innate IFNγ responses to infections, orchestrating both NK and CD8^+^ T cells in this response.^26, 27^ To investigate whether SSM support early IFNγ production by AS01, we depleted SSM by i.m. administration of clodronate liposomes 6 days before the injection of AS01. Clodronate liposomes have been reported to efficiently deplete LN macrophages, without affecting other immune-cell populations at steady state.^28^ The levels of NK cells and CD8^+^ T cells in the dLN were lower with clodronate treatment than with the control treatment in mice injected with PBS or AS01, but the difference was only significant (in a post hoc test) in the mice injected with AS01 (Fig. 6a). The depletion of SSM almost entirely blocked the AS01-induced IFNγ production by lymphoid (CD45^+^) cells (Fig. 6b).

Given that SSM are an essential reservoir of IL-18 and that this cytokine was required for innate IFNγ responses in the dLN upon infection with lymph-borne bacteria,^27^ we investigated its role in our model. Both NK and CD8^+^ T cells in the dLN expressed the receptor for IL-18 (IL-18R), with higher levels observed on NK cells, and no upregulation observed after AS01 injection (Fig. 6c, *top*). NK cells expressed similar levels of IL-18R irrespective of IFNγ production, whereas IFNγ^+^ CD8^+^ T cells expressed higher levels of IL-18R than their IFNγ^−^ counterparts (Fig. 6c, *bottom*). We then investigated whether AS01-induced innate IFNγ production was affected by IL-18 deficiency by using *Il18*^*−/−*^ mice, and by antibody-mediated depletion of IL-12 (αIL-12p40 antibody i.p. 1 day before vaccination), because of the known synergy between IL-12 and IL-18 to promote IFNγ responses. We observed that AS01-induced accumulation of IFNγ^+^ lymphoid cells was dependent on both IL-18 and IL-12 (Fig. 6d). IFNγ^+^ NK-cell levels were reduced in the absence of IL-18 or depletion of IL-12, and further reduced when both cytokines were targeted (Fig. 6e). By contrast, AS01-induced accumulation of IFNγ^+^ CD8^+^ T cells was mostly dependent on IL-18 (Fig. 6f).

Next, we asked whether targeting IL-12 and IL-18 decreased adaptive immune responses to vaccination. Mice were immunized with HBs + OVA/AS01 or unadjuvanted HBs + OVA as in Fig. 3d. Neither IL-12 depletion nor IL-18 deficiency significantly affected the HBs-specific CD4^+^ T-cell response to HBs + OVA/AS01 (Fig. 6g). By contrast, IL-12 depletion together with IL-18 deficiency significantly lowered the HBs-specific CD4^+^ T-cell response, most notably among the (multifunctional) HBs-specific CD4^+^ T cells expressing IFNγ and at least one other cytokine (Supplementary Fig. 5A). Overall, these results suggest that IL-18 and IL-12 contribute to the innate IFNγ response, both individually and in a synergistic fashion. Moreover, they support the hypothesis that these two cytokines synergize in promoting cellular adaptive immune responses to vaccination with AS01-adjuvanted antigens.

### Immunization with an AS01-adjuvanted vaccine is associated with innate IFNγ response across species, and it is associated with protection in a mouse model of malaria vaccination

To evaluate the relevance of our findings in mice with respect to human vaccination, we analyzed the serum IFNγ data from a clinical trial of the candidate malaria vaccine RTS,S/AS01 (Trial NCT00075049^12^). Data were collected at 24 h after the first RTS,S/AS01 dose, a time point in which elevated serum IFNγ was detectable after vaccination in mice (Supplementary Fig. 5B). RTS,S/AS01 vaccination resulted in increased serum levels of IFNγ (Fig. 6h), indicating that an AS01-adjuvanted vaccine elicits an innate IFNγ response in humans. To extend these findings to the dLN, we evaluated vaccine responses in non-human primates, a more clinically relevant species than mice. In these experiments, macaques were vaccinated with AS01-adjuvanted VZV glycoprotein E (VZV) (Fig. 6i, j) or HIV recombinant fusion protein F4 (Fig. 6k). At 24 h, serum levels of IFNγ increased in the majority of AS01-adjuvanted vaccine recipients (Fig. 6i). In the dLN at 24 h, IFNγ levels were significantly higher in recipients of gE/AS01 than recipients of gE alone (Fig. 6j). After 24 h, the levels of IFNγ rapidly subsided in both the sera and dLNs. In addition, at 24 h, the level of CD45^+^ IFNγ^+^ cells was higher in the dLN than non-draining lymph nodes of recipients of gE/AS01, but not in the recipients of gE/alum (Fig. 6k). Overall, these data show that injection of AS01 results in the induction of IFNγ production locally in the dLN in a clinically relevant model. Hence our results suggest that elevated serum concentrations of IFNγ (that can readily be measured in vaccinees) reflect the AS01-related induction of IFNγ^+^ lymphoid cells in the dLN.

Finally, we depleted NK and IFNγ^+^CD8^+^ T cells in the dLN (that express asialoGM1 [AGM1]; Supplementary Fig. 5C) by administering α-AGM1 antibody before vaccination. At 6 h after vaccination with RTS,S/AS01, α-AGM1 treatment resulted in the transient depletion of NK cells and IFNγ-producing CD8^+^ T cells in the dLN, and in decreased IFNγ levels in the serum and dLN (Supplementary Fig. 5D–G and Supplementary Fig. 5I). To evaluate the relevance of our findings to vaccine-induced protection, we used α-AGM1 treatment in a mouse model of malaria vaccination and challenge. C57BL/6 mice were immunized with RTS,S/AS01 (as in Fig. 3d) and then challenged with transgenic *Plasmodium berghei* parasites expressing the full length *Plasmodium falciparum* CSP.^29^ As almost no CSP-specific CD8^+^ T-cell response is elicited by RTS,S/AS01 (Fig. 1c), this model allowed us to minimize the impact of the reported off-target effects of α-AGM1 on adaptive CD8^+^ T cells.^30^ Treatment with α-AGM1 before vaccination with RTS,S/AS01 resulted in a decreased frequency of CSP-specific cytokine-producing CD4^+^ T cells, including polyfunctional CD4^+^ T cells (Fig. 6l, Supplementary Fig. 4J). By contrast, α-AGM1 treatment 3 days after vaccination did not affect CSP-specific CD4^+^ T-cell frequencies (Supplementary Fig. 4H). Treatment with α-AGM1 before vaccination also lowered the titres of full-length CSP-specific IgGs, but levels of IgGs specific for a protective repeat epitope were not significantly affected (Fig. 6m).

In response to malaria challenge, all control mice (mice that did not receive RTS,S/AS01) developed blood-stage malaria by 4 days after challenge. By contrast, of the mice that received RTS,S/AS01 and were not treated with α-AGM1, 30% remained protected from malaria at 12 days after challenge (consistent with human data^13^). In the mice that received RTS,S/AS01 and were α-AGM1-treated, 10% remained protected to day 12 post infection (Fig. 6n). In comparison with control mice, the risk of infection was reduced by RTS/AS01 vaccination alone (hazard ratio = 0.224, 95% CI = 0.077–0.654), but not by RTS/AS01 vaccination and α-AGM1-treatment (hazard ratio = 0.499, 95% CI = 0.194–1.284). Overall, these data support the hypothesis that IFNγ-producing lymphoid cells play an important role in promoting protective immunity after vaccination with RTS,S/AS01.

## Discussion

Combination adjuvants promote potent antigen-specific immunity and increased protection from disease upon vaccination in humans.^1^ The potency of these adjuvants is likely to rely on complex molecular and cellular interplays, which are still poorly understood in vivo. Here, we apply a customized statistical framework to unravel the way that MPL and QS-21 contribute to the transcriptional response to AS01 in the dLN. We also show that MPL and QS-21 contribute to the synergistic production of innate IFNγ at the earliest stages of vaccination. This IFNγ production is critical for promoting polyfunctional Th1-biased antigen-specific CD4^+^ T-cell responses to AS01-adjuvanted antigens and also impacts on the quality of the antibody response with a reduction in Th1-isotype switching. IFNγ is mainly produced by activated NK and CD8^+^ T cells, and the process is controlled by the SSMs and IL-12/IL-18, similar to the response to pathogens.^27, 31^ We also confirmed the clinical relevance of these findings by showing that IFNγ production can be detected in the serum of vaccine recipients at 24 h after vaccination with RTS,S/AS01, and in the dLN of NHPs at 24 h after vaccination with AS01-adjuvanted antigens. Finally, we show that innate IFNγ production is important in mediating protection in a mouse model of vaccination against malaria. Altogether, the study provides new insights into how the unique design of AS01 (that is, the specific combination of QS-21 and MPL in AS01) can account for the efficacy of AS01-containing vaccines.

The rationale behind Adjuvant Systems and combination adjuvants is that combining two immunostimulants may trigger multiple immune pathways and improve the protective response to vaccination.^1, 15^ Yet, so far, most analyzes of combination adjuvants relied on visual comparisons between components in the absence of comprehensive statistical testing.^20^ Here, we used a customized taxonomy and statistical framework to understand how MPL and QS-21 interact in AS01 at the transcriptional level. In using this approach, we observed complex interplays, in addition to the expected additive or specific effects related to each immunostimulant. Noticeably, 13–15% of differentially expressed genes were regulated in an antagonistic fashion, suggesting interference between MPL and QS-21 downstream processes in vivo. Moreover, 1–3% of the transcriptional response was classified as emergent. This highlights the complexity of the response to adjuvant systems, with new, not pre-existing properties emerging from the combination of different immunostimulants. Emergent genes contributed the most to the variation in gene expression induced by AS01, suggesting an important role of these genes in mediating AS01’s adjuvanticity.

It is well established that IFNγ plays a crucial role in the innate immune response to infection, and that innate IFNγ can promote a Th1- polarizing environment in vivo.^16, 32, 33^ However, the role of innate IFNγ in clinically relevant adjuvant responses has not been investigated. We report that the combinatorial nature of AS01 promotes the production of IFNγ by lymphoid cells in the dLN in the first few hours after vaccination. NK cells and CD8^+^ T cells were the main sources of innate IFNγ. IFNγ^+^ NK cells were mainly resident in the dLN, as little recruitment of NK cells was observed in the first hours after vaccination. CD8^+^ T cells had features of virtual memory CD8^+^ T cells, which have been shown to participate in innate immune responses in an antigen-independent fashion.^34^ Both the NK and the CD8^+^ T-cell responses were dependent on SSMs, in a mechanism involving IL-18 and IL-12, and independent from monocyte recruitment in the dLN, as the responses were preserved in *Ccr2*^−/−^ mice (Kaat Fierens and Bart Lambrecht, personal communication). The innate IFNγ response to AS01 was remarkably similar to that described following infection of mice with both extracellular and intracellular pathogens.^27^ These results suggest that in its induction of potent adaptive responses to the vaccine antigen, AS01 targets the complex prepositioning of cells in the dLN that are poised to limit bacterial spread during an infection. Given the complexity of AS01-mediated modulation of DC function and the heterogeneous population of cells in the dLN,^7^ further experiments are needed to detail the interaction between NK cells and myeloid cells in dLN. Interestingly, a recent report showed early IFNγ production in the dLN of mice immunized with glucopyranosyl lipid adjuvant-stable emulsion (GLA-SE).^35^ Although the levels observed in that study were lower than those we report for AS01, it should be noted that GLA-SE is also able to induce T-cell responses in vivo,^35^ supporting the hypothesis that early IFNγ production is important for obtaining productive T-cell responses to adjuvanted vaccines.

The adjuvanticity of AS01 is at least partially mediated by the recruitment and activation of CD11c^+^ MHCII^Hi^ DCs.^7^ Here, we show that the maturation of DCs is dependent on innate IFNγ release (presumably via a cell-intrinsic mechanism^36^), and that IFNyR deficiency or simultaneous impairment of both IL-12 and IL-18 results in defective CD4^+^ cellular immune responses to vaccination. In addition, mice specifically deficient in NK cells developed reduced CD4^+^ T-cell responses to vaccination. Overall, this indicates that innate IFNγ is required to induce CD4^+^ T-cell responses, with a Th1-bias after immunization with an AS01-adjuvanted vaccine, as further supported by the reduction in IgG2c antigen-specific antibodies in the absence of innate IFNγ signals. However, the CD8^+^ T-cell response and the overall magnitude of the humoral immune response were mostly preserved in all these models suggesting that other yet undiscovered mechanisms also contribute to AS01 adjuvanticity. Nevertheless, the depletion of IFNγ-producing cells resulted in decreased RTS,S-mediated protection in a malaria challenge model, accompanied by a decreased CSP-specific CD4^+^ T-cell response and limited effects on protective antibodies. This suggests that the modulation of adaptive immunity mediated by innate IFNγ is relevant to protection. In addition, it suggests some role for both cellular and humoral adaptive immunity in RTS,S-mediated protection from malaria, as hinted by findings from clinical trials.^12^

As QS-21 induces inflammasome activation in vitro and in vivo^37^ and MPL induces IL-12 secretion in vitro,^8^ we hypothesized that the combination of these two immunostimulants in AS01 is essential for early IFNγ production. Accordingly, the innate IFNγ response to AS01 was fully dependent on both IL-18 and IL-12 production in the dLN. Other members of the inflammasome-dependent cytokines, such as IL-1β, might contribute to the IFNγ response in vivo, but are likely to be redundant with IL-18. Furthermore, the components of AS01 reach the dLN as early as 30 min after immunization^7^ (and data not shown). The liposome formulation of AS01 is most likely to play a role in its rapid drainage to the dLN and activation of SSM, as liposomes can improve the targeting of their components to the lymph node^38^ and promote uptake by macrophages.^39^

We identified innate IFNγ as a major player in the innate immune response to AS01 in the earliest stages after immunization. Although the role for adaptive, T-cell-derived IFNγ in promoting effective immunization is well established,^40^ little information is available regarding the role of this cytokine in the early response to vaccination. Yet, evidence from clinical studies suggests that major cellular and molecular changes occur in the first 24 hours after immunization in humans.^41–43^ We show that vaccination with AS01-adjuvanted antigens results in upregulation of IFNγ levels in the serum of human subjects and in the serum and dLN of NHPs. Although caution should be used when extending the findings from animal models to humans, this latter observation supports the hypothesis that innate cells underlie IFNγ production in humans who receive an AS01-adjuvanted vaccine. Indeed, CD56^Hi^CD16^–^ NK cells are enriched in human lymph nodes and are more efficient at producing IFNγ than peripheral-blood NK cells,^44, 45^ and CD8^+^ T cells with innate features were recently described in human blood and cord blood samples.^46^ More investigations in appropriate models such as NHPs will be needed to understand the contribution of these cell types to innate immune response to vaccination. Interestingly, individuals vaccinated with RTS,S/AS01 and protected from malaria presented increased activation of an IFN-related blood transcriptional module at D1 post vaccination, compared with non-protected individuals.^47^ However, further work will need to fully demonstrate the requirement of an innate IFNγ response for the induction of effective vaccination in humans. It is also possible that such a mechanism will be amplified with successive vaccine doses, because antigen-specific memory-T cells can promote IFNγ production by innate immune cells.^36^ Indeed, a positive feedback loop between NK cells and CD4^+^ T cells takes place during vaccination, malaria and VZV infections.^48–50^

Overall, our study provides compelling information regarding the mechanism of action of AS01, an adjuvant which has proven successful in improving immunogenicity and efficacy in vaccines for challenging diseases such as malaria and herpes zoster.

## Materials and methods

### Vaccine formulations

All antigens (HBs, OVA, CSP, VZV glycoprotein E, all clinical-grade) and the Adjuvant System AS01 were produced at GSK. Unless otherwise stated in the figure legends, AS01 contained 100 μg/ml 3D-MPL (GSK), 100 µg/ml QS-21 (*Quillaja saponaria* Molina, fraction 21, licensed by GSK from Antigenics LLC, a wholly owned subsidiary of Agenus Inc., a Delaware, USA corporation) and was formulated in liposomes. In experiments with RTS,S/AS01, a variant of AS01 (AS01_E_ = 50 μg/ml 3D-MPL (GSK), 50 µg/ml QS-21) was used. Set-up experiments showed no impact of lower doses of AS01 on its adjuvanticity in mice (data not shown).

### Human studies

All blood samples were obtained from the RTS,S vaccine clinical trial (ClinicalTrials.gov NCT00075049).^12^ The clinical trial was conducted in accordance with all applicable regulatory requirements, including the Declaration of Helsinki (1996). The clinical trial was approved by institutional review boards from the Walter Reed Army Institute of Research (WRAIR) Human Use Review Committee (HURC) and the United States Army Medical Research and Materiel Command (USAMRMC) Human Subjects Research Review Board (HSRRB). Written-informed consent was obtained from each participant prior to the performance of any study-specific procedures in accordance with relevant ICH Guidelines, US Army Regulations and principles of the Declaration of Helsinki. Serum IFNγ levels at D0 and D1 after immunization were measured by ELISA (R&D Systems) in 33/35 trial participants receiving RTS,S/AS01 due to missing data in 2 individuals.

### Animal immunizations

Animal husbandry and experiments were ethically reviewed and carried out in accordance with European Directive 2010/63/EU and the GlaxoSmithKline Biologicals S.A. Policy on the Care, Welfare and Treatment of Animals. For the malaria challenge model (conducted at WRAIR) experiments were conducted under an approved animal use protocol in an AAALACi accredited facility in compliance with the Animal Welfare Act and other federal statutes and regulations relating to animals and experiments involving animals and adheres to principles stated in the Guide for the Care and Use of Laboratory Animals, NRC Publication, 2011 edition.

C57Bl/6 mice were purchased from Harlan Horst, Netherlands. *Ifngr*^*−/−*^, *Il18*^*−/−*^ and *ROSA-stop*^*flox*^*−DTA* mice were purchased from Jackson Laboratories (USA). Nkp46(iCre) mice were a gift of Prof. Eric Vivier. Mice were allocated across experimental groups by random draw. Experimenters were not blinded during tests. The i.m. injections were performed in 6–8 week-old mice, in both hind limbs, in the *gastrocnemius* (gcm) muscles and in a volume of 50 µl/muscle, unless otherwise stated. Iliac lymph nodes were identified as draining in set-up experiments, which also showed minimal immune activation in non-draining lymph nodes. Anti-IL-12p40 mAb (C17.8, 0.5 mg i.p.) was purchased from BioXCell and injected i.p. the day before immunization. Anti-IFNγ mAb (XMG1.2) (BioXCell), was injected i.p. 48 h before immunization. Clodronate liposome and control liposomes (Clodrosome) were injected i.m. (2 × 20 μl) in the gcm muscles 6 days before immunization. For NHP studies, 4–6 years old Chinese rhesus macaques were used. Animals had been previously used in unrelated vaccination studies, but a resting period of 6 months minimum was imposed to minimize any impact on this study. NHPs were randomized and immunized intramuscularly in both left and right biceps with AS01 (250 μl/injection) and VZV glycoprotein E or HIV recombinant protein F4. Axillary lymph nodes were used as dLNs and cervical lymph nodes as non-draining lymph nodes.

### Malaria challenge model

Mice were challenged i.v. on Day 0 with 3000 transgenic *P. berghei* sporozoites expressing *P. falciparum* CSP, collected from a single mosquito lot.^29^ Mice were monitored from Day 2–12 for parasitemia by blood smear under microscope. Mice were considered infected and euthanized after exhibiting parasitemia on two consecutive days. All mice were euthanized on Day 12.

### Microarray analysis

Total RNA was isolated by homogenizing pooled dLN in Tripure reagent (1 ml/100 mg tissue; Roche Applied Science) and then extracted with chloroform followed by RNeasy Minikit (Qiagen) according to the manufacturer’s protocol. A DNAse treatment was applied on the RNeasy column to avoid genomic DNA contamination. RNA was concentrated by ethanol precipitation, and quantified by RiboGreen (Life Technologies). 1 µg of each RNA sample was used for target preparation, using a one-cycle cDNA synthesis kit, and hybridized to GeneChip Whole Mouse Genome 430 2.0 arrays (Affymetrix). Data acquisition was performed using GeneChip Operating Software (Affymetrix). Data were quality controlled and normalized (RMA normalization) in R using Bioconductor packages. The data analysis pipeline is presented in Supplementary Fig. 6. Linear modeling and contrast analysis were performed using the limma R package, PCA plots were generated using pca3d. Enrichment analysis on PCA components were generated using the tmod and tagcloud packages. Heatmaps were generated using the pheatmap packages. For identification of interplay between AS01 components, for each gene, an effect score over PBS was calculated. The type of interplay for each DEG was identified in R using the algorithm described in ref.^15^ Briefly, an empirical Bayes procedure (in limma R package) was used to estimate the contrasts that make up the different forms of interplay and to assess their significance. The significance of a particular form of interplay was subsequently obtained by applying an intersection-union hypothesis testing procedure to the proper combination of contrasts. Equivalence intervals of [−1,1] and a *p*-value of <0.05 were used as cutoff. Process Network enrichment analysis was performed in MetaCore (Thomson Reuters).

### Code availability

R code with an implementation of the procedure to identify different forms of interplay between AS01 components is included in the Supporting Information of ref.^15^

### Determination of antigen-specific T-cell and antibody responses to vaccination

Antigen-specific antibody concentrations in the serum (defined by internal standards) were measured by ELISA, as previously described.^7, 13^ To assess antigen-specific T-cell response, 10^6^ splenocytes from immunized mice were stimulated in vitro with peptide pools of 15-mers with 11 amino acids overlap (1 µg/ml). Anti-CD49d and anti-CD28 antibodies (1 µg/ml, BD Biosciences) were added to the culture and the cells were incubated 2 h at 37 °C 5% CO_2_ in RPMI 10% FCS + additives (l-Glutamine (2 mM), Pennicilin/Streptomycin (100 U/100 µg), sodium pyruvate 1 mM and MEM non-essential amino acids (1 × ) (all from Invitrogen)). Brefeldin A (BFA) (1 μg/ml, BD Biosciences) was then added and cells were further cultured overnight at 37 °C 5% CO_2_. Cell staining was performed as described previously.^7, 13^ Briefly, cells were washed and stained for surface markers, then fixed and permeabilized using the BD Cytofix/Cytoperm solution kit. Cells were then stained with αIL-2 FITC, anti-IFNγ-APC, and anti-TNF-α-PE (all from BD) for 2 h at 4 °C. The samples were analyzed with the LSRII flow cytometer (BD) and FlowJo software. A complete list of antibodies used in this study is presented in Supplementary Table 1.

### Innate cell phenotyping

dLNs were first treated by mechanical dissociation in 3 ml RPMI medium containing DNase I (100 µg/ml, Roche), 1 % FCS and Liberase (Roche) at 0.26 U/ml for 30 min under agitation at room temperature. Digestion was stopped by adding 10 mM EDTA and incubating on ice. Cells were filtered (100 µM nylon cell strainer, BD Biosciences), washed and resuspended in PBS + 2 mM EDTA + 2 % FCS. The analysis of innate cell phenotype was performed as previously described.^7^ Monocytes were defined as Ly6C^high^ CD11b^+^,Ly6G^−^ cells and neutrophils were defined as SSC^high^, CD11b^+^, Ly6G^high^. After exclusion of neutrophil, monocyte, and lymphocyte populations, DCs were gated as CD11c^mid^MHCII^high^. For identification of IFNγ producing cells in mice, dLNs were dissociated and filtered cells were incubated for 3 h in RPMI 10% FCS + additives in the presence of BFA (1 µg/ml, BD Biosciences) and Monensin (1 µg/ml, BD Biosciences) at 37 °C 5% C0_2_. After treatment with 2.4G2 antibody for 10 min to block the Fc receptor, cells were stained for the following markers: NK1.1, TCRβ, TCRγδ, CD45, CD8, CD49b, CD44, CD27, CD69, CD25, CD4, RORγ, EOMES, CXCR3, IL-18R, and CD122 using antibodies from BD Bioscience or eBiosciences. For NHP studies, collagenase-treated dLN cell suspensions were incubated for 5 h in RPMI 10% FCS + additives in the presence of BFA (1 µg/ml, BD Biosciences) and Monensin (1 µg/ml, BD Biosciences) at 37 °C 5% C0_2_. Cells were then stained for the following surface markers: CD20, CD177, NKp46, CD45, CD11c, CD123, CD8, CD11b, CD3, CD19, CD16, CD14, HLADR, CD4, and TCRγδ. Cells were then fixed and permeabilized using the BD Cytofix/Cytoperm solution kit and stained with anti-IFNγ APC, FITC or PeCy7.

### Analysis of cytokine levels

For the assessment of pro-inflammatory cytokine concentration, dLNs were collected after immunization at the indicated time points and stored at −80 °C until further analysis. Organs were homogenized and cytokines were measured in supernatants as described previously.^7^ Protein levels were measured by Cytokine Bead Analysis (BD Biosciences) or ELISA (R&D system) (mouse studies) or multiplex assay (Luminex,Milliplex Kit, Millipore) (NHP studies). For qPCR analysis, RNA was isolated by homogenizing dLN in Tripure reagent (Roche Applied Science) and then extracted with chloroform followed by RNeasy Minikit (Qiagen) according to the manufacturer’s protocol, with DNAse treatment. RNA was concentrated by ethanol precipitation, and quantified by RiboGreen (Invitrogen). 500 ng of total RNA were reverse-transcribed using Superscript II RNase H (Invitrogen) as per manufacturer’s protocol. RNA expression was quantified by real time PCR (RT-PCR) using the ABI SYBR Green Mastermix (Applied Biosystems) (primers sequence for IFNγ [5′-GCATTCATGAGTATTGCCAAGTTTG-3′, 5′-CGGATGAGCTCATTGAATGCTT-3′], for HPRT [5′-GCAAACTTTGCTTTCCCTGG-3′, 5′-ACTTCGAGAGGTCCTTTTCACC-3], primer efficiency 90–110%). RT-PCR was run the ABI Prism 7900HT Sequence Detection System (Perkin Elmer). Expression levels were quantified using the comparative Ct method and gene expression was determined relative to the mean value of the matched PBS-treated controls.

### Statistical analyzes

Sample size with adequate statistical power in animal studies was determined based on previous experiments. Where appropriate, data were log-tranformed and assumed to have a normal distribution. Depending on the study design, a Student's *t*-test or an ANOVA (one- or two-way, depending on the experimental design) followed by Bonferroni corrected *t-*test were applied to identify differences between treatments. Only significant differences are reported on the graphs. Two-way ANOVA was performed to assess experiments with a 2 × 2 factorial design (for instance, when mice from 2 different strains were vaccinated with either AS01 or PBS + Ag). When 2- way ANOVA is performed, the interaction test addresses the question whether the effect of treatment (for instance vaccination) is the same in both conditions tested (for instance 2 different mouse strains). If the *p-*value is < 0.05, then the treatment has different effects in the two conditions. Statistical significance: *p < 0.05, **p < 0.01, ***p < 0.001, ****p < 0.0001.

### Data availability

Transcriptomics data are available on GEO database with access number GSE98322. Preclinical data that support the findings of this study are available from GSK Vaccines but restrictions apply to the availability of these data. Data are, however, available from the authors upon reasonable request and with permission of GSK Vaccines. The results summary for the clinical study (GSK study number 257049/027 - NCT00075049) is available on the GSK Clinical Study Register and can be accessed at www.gsk-clinicalstudyregister.com. For interventional studies that evaluate our medicines, anonymized patient-level data will be made available to independent researchers, subject to review by an independent panel, at www.clinicalstudydatarequest.com within 6 months of publication. To protect the privacy of patients and individuals involved in our studies, GSK does not publically disclose patient-level data.

## Electronic supplementary material

Supplementary material
