# Author Correction: Cellular and molecular synergy in AS01-adjuvanted vaccines results in an early IFNγ response promoting vaccine immunogenicity

**DOI:** 10.1038/s41541-018-0047-7

**Published:** 2018-03-21

**Authors:** Margherita Coccia, Catherine Collignon, Caroline Hervé, Aurélie Chalon, Iain Welsby, Sophie Detienne, Mary J. van Helden, Sheetij Dutta, Christopher J. Genito, Norman C. Waters, Katrijn Van Deun, Age K. Smilde, Robert A. van den Berg, David Franco, Patricia Bourguignon, Sandra Morel, Nathalie Garçon, Bart N. Lambrecht, Stanislas Goriely, Robbert van der Most, Arnaud M. Didierlaurent

**Affiliations:** 1 GSK Vaccines, Rixensart, Belgium; 2 Institute for Medical Immunology, Université libre de Bruxelles, Gosselies, Belgium; 3 Vlaams Instituut voor Biotechnologie, Inflammation Research Center, Ghent University, Ghent, Belgium; 4 Malaria Vaccine Branch, Military Malaria Research Program, Walter Reed Army Institute of Research, Silver Spring, Maryland USA; 5 Department of Methodology and Statistics, Tilburg University, Tilburg, The Netherlands; 6 Department of Psychology, Katholieke Universiteit Leuven, Leuven, Belgium; 7 Biosystems Data Analysis, Swammerdam Institute for Life Sciences, University of Amsterdam, Amsterdam, The Netherlands

**Correction to:** *npj Vaccines* (2017). 10.1038/s41541-017-0027-3; Published 8 September 2017

In the original version of the article, Fig. 2b was mislabelled. The second row was incorrectly labelled as “Mi_MPL_ > Mi_PBS_ and Mi_QS_ ≈ M_PBS_” this has now been corrected to “Mi_QS-21_ > Mi_PBS_ and Mi_MPL_ ≈ Mi_PBS_”. The third row was incorrectly labelled as “Mi_QS21_ > Mi_PBS_ and Mi_MPL_ ≈ Mi_PBS_” this has now been corrected to “Mi_MPL_ > Mi_PBS_ and Mi_QS-21_ ≈ Mi_PBS_”. These errors have now been corrected in the PDF and HTML versions of this article.

**Fig. 2b:**
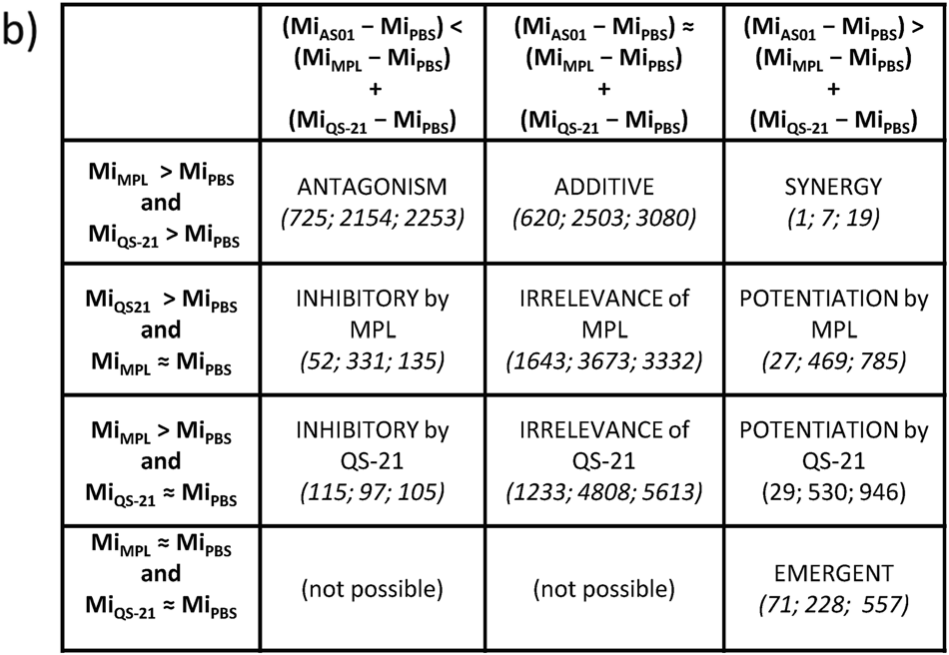
A taxonomy of possible interplays between QS-21 and MPL in AS01 is shown. The conditions to be met for each category are represented in bold. *M*i represents the gene expression level for each gene i. Numbers in italics represent the number of AS01 DEGs belonging to each category at 2 h, 4 h, and 6 h after immunization, respectively

