# Supplementary material for: Cellular and molecular synergy in AS01-adjuvanted vaccines results in an early IFNγ response promoting vaccine immunogenicity
Source: NPJ Vaccines. 2017 Sep 8;2:25. doi: 10.1038/s41541-017-0027-3 (PMC5627273; doi:10.1038/s41541-017-0027-3)
Supplement: Supplementary file 1 — Supplementary material [file 41541_2017_27_MOESM1_ESM.docx]

**SUPPLEMENTARY MATERIAL**

**
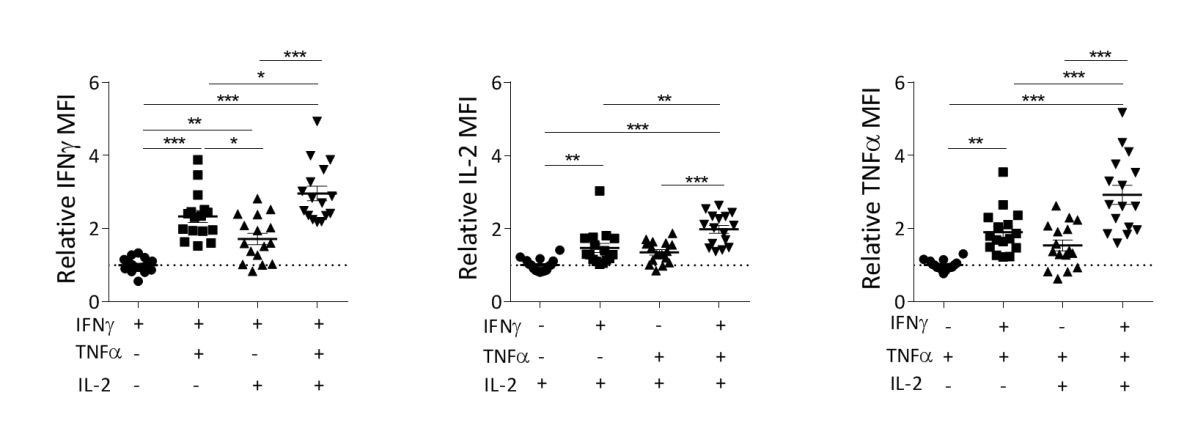
Supplementary Figure 1.** C57BL/6 mice were immunized with AS01 and HBs (HBs 100 μg/ml, 2 × 50 μl/injection i.m.) on day 0 and day 14. On day 21, spleens were harvested and splenocytes were restimulated with a pool of peptide spanning the length of the utilized antigen and analysed by intracellular staining (ICS). Data show relative mean fluorescence intensity (MFI) for different cytokines (MFI single cytokine producers=1) in CD4^+^ T cells. Results from 2 pooled experiments are shown. Data were analysed using one-way ANOVA followed by Bonferroni post-hoc test to compare all groups. *p<0.05, **p<0.01, ***p<0.001. Data are shown as mean ± SEM.


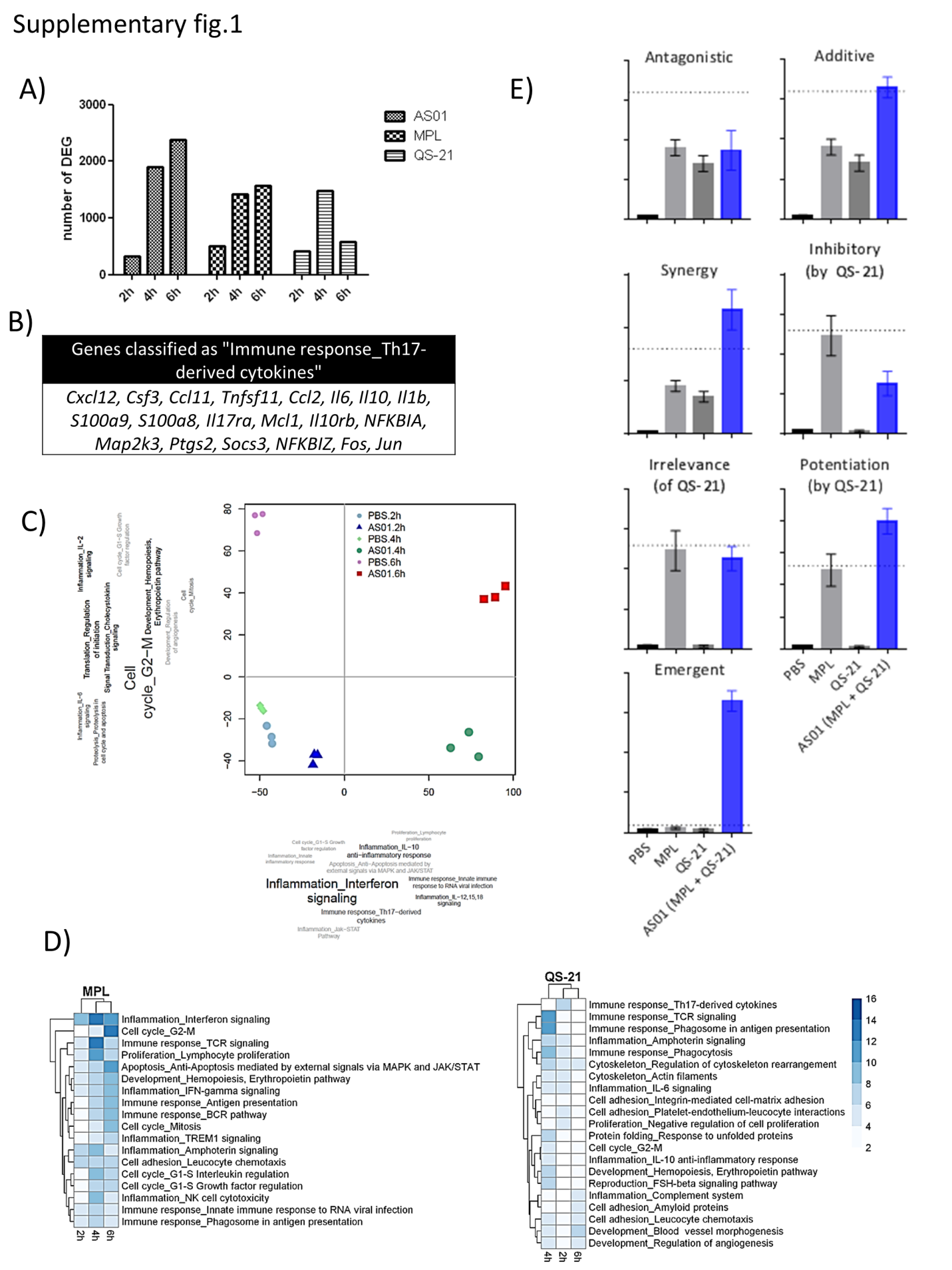


**Supplementary Figure 2.** C57BL/6 mice (n = 3/adjuvant/time point) were immunized once with HBs + AS01=MPL+ QS-21, MPL= 50 μg/ml, QS-21= 50 μg/ml, 2 × 50 μl injections, i.m. dLN were isolated at indicated time points and gene expression was assessed by microarray analysis. Linear models and contrast analysis was used to identify differentially expressed genes (DEG, cut-offs: Fold Change FC >2, p.value for contrast <0.01, adjusted for multiple comparisons). A) Number of DEG obtained by limma analysis for each adjuvant. B) Genes classified as "Immune response_Th17-derived cytokines" in the dLN of mice immunized with AS01 2 h post injection.C) PCA was used to reduce the dimensionality of the DEG list. PC1 and PC2 are represented here, together with the scores of each sample. Word Clouds represent the functional pathways enriched in each PC’s loadings. The size of the character is proportional to the –log(p value) of the enrichment, while the intensity of the colour is proportional to the effect size. D) C57BL/6 mice (n = 3/adjuvant/time point) were immunized once with HBs + AS01 or MPL or QS-21 or PBS (AS01=MPL+ QS-21, MPL= 50 μg/ml, QS-21= 50 μg/ml, 2 × 50 μl injections, i.m, all adjuvants formulated in liposomes). dLN were isolated at indicated time points and gene expression was assessed by microarray analysis. Results from AS01-immunised mice are shown in Fig.2A.E) Examples of different interplays between adjuvants.


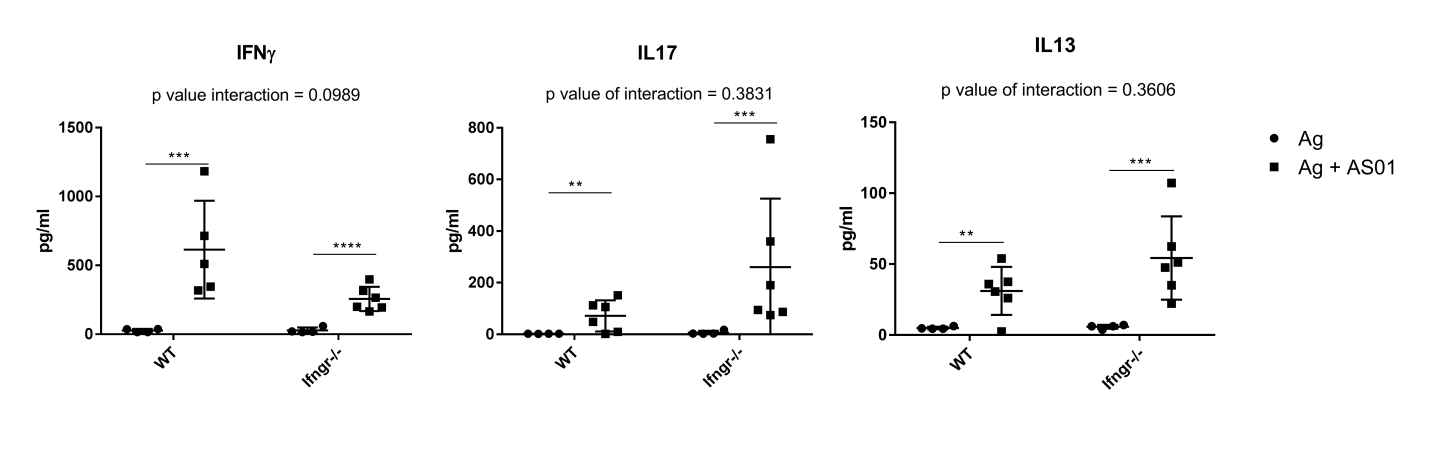


Supplementary Figure 3. C57BL/6 or Ifngr-/- mice were immunized with AS01 and HBs/OVA (2 × 50 μl/injection i.m.) twice at a 2 week interval. Mice were sacrificed on D21 and splenocytes were restimulated with a pool of peptide spanning the length of HBs. After overnight culture, supernatants were harvested and analysed by Cytokine Beads Assay. Data are shown as mean ±SD. Data were analysed by 2-way ANOVA followed by Bonferroni-corrected t test.

**
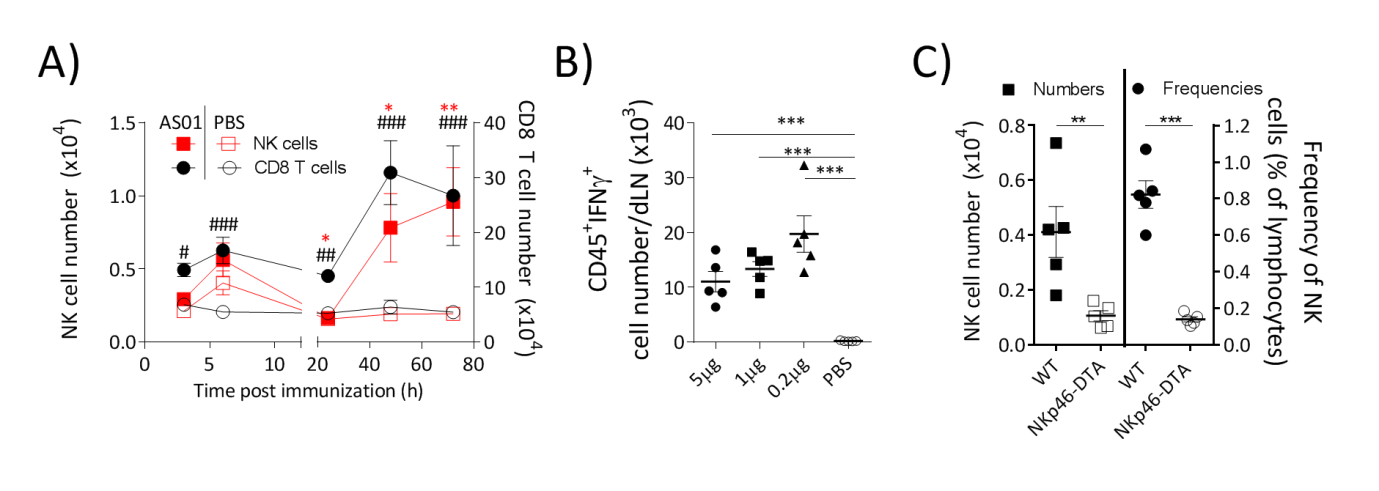
**

**Supplementary Fig. 4.** A) C57BL/6 mice were treated as in 5A and the accumulation of NK and CD8 T cells in the dLN at indicated time points was assessed (n=4-8 from 1 of 2 independent experiments). * indicate level of significance for NK cells, # for CD8 T cells. B) C57BL/6 mice were immunized with AS01 or its components at indicated doses. After 6 h, dLN were harvested and the number of IFNγ^+^ CD45 cells was evaluated by FACS. C) Number and frequency of NK cells in the dLN of NKp46-DTA or WT mice at steady state.

**Supplementary Figure 5.** A) C57BL/6 WT or *Il18-/-* mice were treated with αIL-12p40 (C17.8, 0.5 mg, i.p.) and immunized the next day with AS01 + HBs/OVA (HBs 80 μg/ml, OVA 20 μg/ml,, 2 x 50 μl/injection i.m.). Mice were immunized twice at 14 days intervals. On day 21, spleens were harvested and splenocytes were restimulated with a pool of peptide spanning the length of HBs and analysed by ICS. The frequency of antigen-specific CD4^+^ cytokine-producing species is shown here. B) C57BL/6 mice were immunized once with HBs + AS01 as above and levels of IFNγ in the serum were tested at indicated time points (p.i., post-immunization). Data from 3 pooled experiments are shown. C) C57BL/6 mice were immunized with AS01 + HBs (AS01=MPL+ QS-21, MPL= 50 μg/ml, QS-21= 50 μg/ml, HBs=100 μg/ml). Six hours later, dLN were harvested and the expression of asialo-GM1 by IFNγ producing NK cells (top) and CD8 T cells was evaluated by FACS. Representative plots are shown (n=5). D-G) C57BL/6 mice were immunized with AS01 + HBs as above, but α-AGM Ab was administered at day -2 and 0. Six hours after immunization, dLN were harvested and analysed by FACS. D) NK cells. E) CD8+ T cells. F) IFNγ+ CD8+ T cells. G) IFNγ+ lymphoid cells (left) and IFNγ levels in the dLN (right). H) Mice were immunized with RTS,S/AS01 according to the schedule in Fig.3D, and received α-AGM Ab 3 days after the last immmunization. Two weeks after the last immunization, CSP-specific CD4 responses were assessed ex vivo. I-J) Mice were immunized with RTS,S/AS01 according to the schedule in Fig.3D, and received α-AGM Ab 2 days before and on the day of immunization. Immune responses were analysed 2 weeks after the last immunization I) Number of NK and T cells in the dLN as assessed by FACS. J) Frequency of cytokine-producing antigen-specific CD4^+^ T cells as analysed by ICS.

**
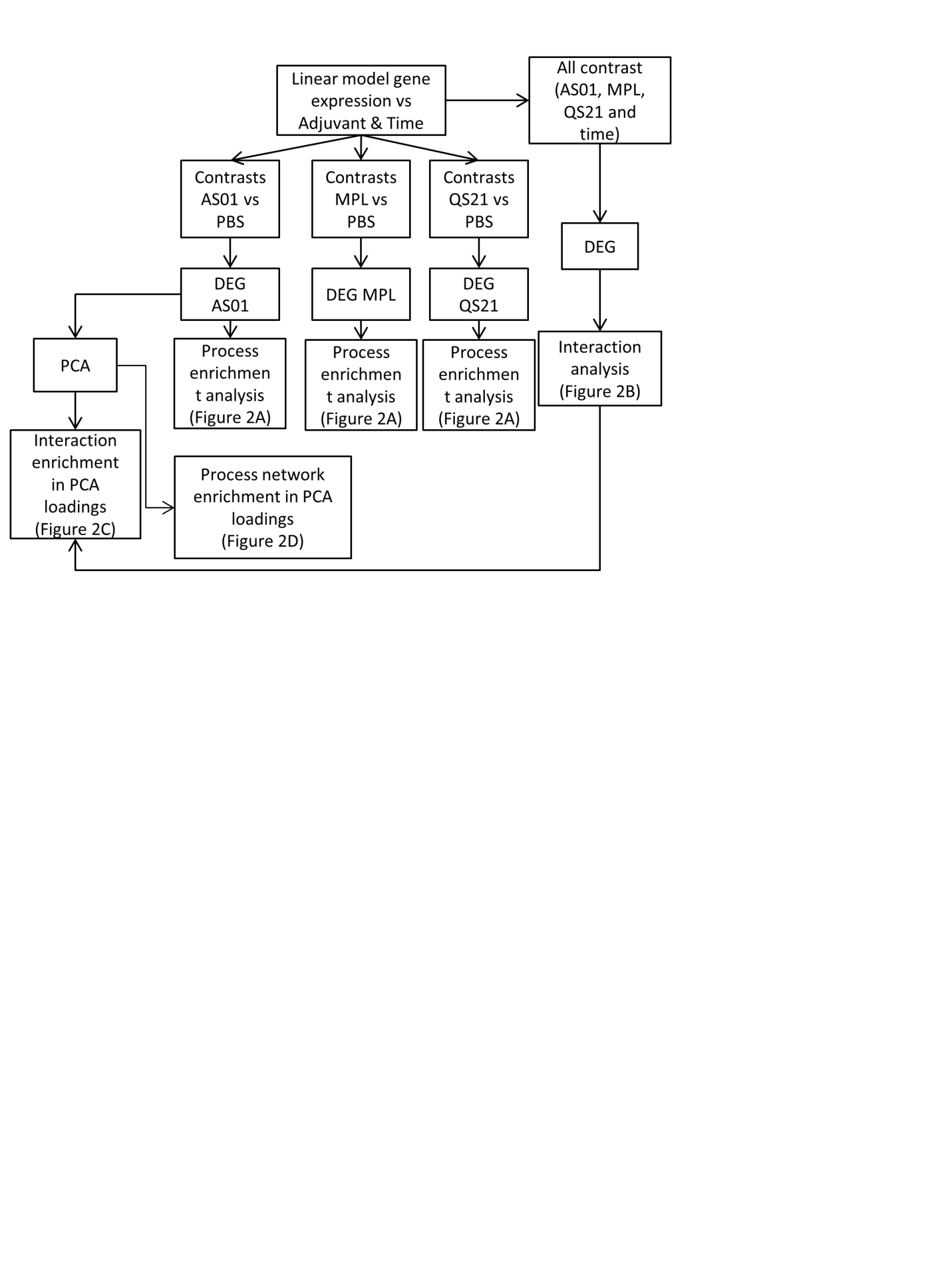
**

**Supplementary Figure 6.** Workflow of data analysis for microarray data.

**Supplementary Table 1.** List of antibodies used across all studies. Depleting antibodies are mentioned in Materials and Methods.

| **Target** | **Reagent/ Target Antigen** | **Conjugation** | **Catalogue Number** | **Supplier** |
| --- | --- | --- | --- | --- |
| Mouse | AsialoGM1 | AF647 | 146003 | Biolegend |
|  | CD11b | PB | RM2828 | Life Technologies |
|  | CD11b | BV650 | 101239 | Biolegend |
|  | CD11b | BV785 | 101243 | Biolegend |
|  | CD11c | PE-CY7 | 25-0114-82 | eBioscience |
|  | CD122 | e450 | [48-1222-82](https://www.thermofisher.com/antibody/primary/query/cd122) | eBioscience |
|  | CD127 | PE | 12-1271-82 | eBioscience |
|  | CD16/32 | NA | 553142 | BD Biosciences |
|  | CD19 | BV510 | 115545 | Biolegend |
|  | CD19 | APC-CY7 | 115530 | Biolegend |
|  | CD19 | BV421 | 115538 | Biolegend |
|  | CD25 | Pe-cy7 | [552880](https://www.bdbiosciences.com/eu/applications/research/t-cell-immunology/regulatory-t-cells/surface-markers/mouse/pe-cy7-rat-anti-mouse-cd25-pc61/p/552880) | BD Biosciences |
|  | CD27 | PerCP | 563603 | BD Biosciences |
|  | CD28 | NA | 553294 | BD Biosciences |
|  | CD3e | BV510 | 100233 | Biolegend |
|  | CD4 | E450 | 560468 | BD Biosciences |
|  | CD4 | PB | 100428 | Biolegend |
|  | CD4 | PE-CY7 | 25-0042-82 | eBioscience |
|  | CD40 | APC | 558895 | BD Biosciences |
|  | CD44 | PerCP-CY5.5 | 45-0441-82 | eBioscience |
|  | CD44 | AF700 | 103025 | Biolegend |
|  | CD45 | BV605 | 563053 | BD Biosciences |
|  | CD49b | PE | 553858 | BD Biosciences |
|  | CD49d | PE | [553157](http://www.bdbiosciences.com/us/applications/research/stem-cell-research/mesenchymal-stem-cell-markers-bone-marrow/mouse/positive-markers/pe-rat-anti-mouse-cd49d-r1-2/p/553157) | BD Biosciences |
|  | CD49d | NA | 553313 | BD Biosciences |
|  | Cd62L | AF700 | 104426 | Biolegend |
|  | Cd62L | FITC | 553150 | BD Biosciences |
|  | Cd62L | e450 | [48-0621-82](https://www.thermofisher.com/antibody/primary/query/CD62L) | eBioscience |
|  | CD69 | FITC | [561929](http://www.bdbiosciences.com/eu/applications/research/t-cell-immunology/regulatory-t-cells/surface-markers/mouse/fitc-hamster-anti-mouse-cd69-h12f3/p/561929) | BD Biosciences |
|  | CD69 | PE | 12-0691-82 | eBioscience |
|  | CD80 | PE | 553769 | BD Biosciences |
|  | CD86 | BIOTIN | 553690 | BD Biosciences |
|  | CD86 | FITC | 553691 | BD Biosciences |
|  | CD8a | PerCP-cy5.5 | 551162 | BD Biosciences |
|  | CD8a | Alexa 700 | 56-0081-82 | eBioscience |
|  | CD8a | PE | 551162 | BD Biosciences |
|  | CD90.2 | PE | 553006 | BD Biosciences |
|  | CXCR3 | BV605 | 126523 | Biolegend |
|  | EOMES | PE | [12-4875-82](https://www.thermofisher.com/antibody/primary/query/eomes) | eBioscience |
|  | IFNg | APC | 554413 | BD Biosciences |
|  | IFNg | PE-CY7 | 25-7311-82 | eBioscience |
|  | IFNg | e450 | 11-7311-82 | eBioscience |
|  | IL-18R | FITC | FAB1216F | R&D Systems |
|  | IL-2 | FITC | 554427 | BD Biosciences |
|  | Live and Dead | Near-IR | L10119 | Thermo Fisher |
|  | Live and Dead | Aqua | L34957 | Thermo Fisher |
|  | Live and Dead | Yellow | L34959 | Thermo Fisher |
|  | Ly6C | FITC | 553104 | BD Biosciences |
|  | Ly6C | BV605 | 563011 | BD Biosciences |
|  | Ly6G | BV711 | 563979 | BD Biosciences |
|  | LY6G | PerCP-CY5.5 | 560602 | BD Biosciences |
|  | MHCII | AF700 | 56-5321-82 | BD Biosciences |
|  | mouse Ig Biot | Biotin | E0413 | Dako Agilent technologies |
|  | NK1.1 | APC | 17-5941-82 | eBioscience |
|  | NK1.1 | PE | 553165 | BD Biosciences |
|  | RORC | PE | [61-6981-82](https://www.thermofisher.com/antibody/primary/query/ror) | eBioscience |
|  | Siglecf | PE | 552126 | BD Biosciences |
|  | STREPTAVIDIN | PerCP | 554064 | BD Biosciences |
|  | TCRb | PerCP-CY5.5 | 45-5961-82 | eBioscience |
|  | TCRb | FITC | 11-5961-85 | BD Biosciences |
|  | TCR-gd | e450 | 48-5711-82 | eBioscience |
|  | TNFa | PE | 554419 | BD Biosciences |
| Non-Human-Primate | Cd11b | APC-Cy7 | 557754 | BD Biosciences |
|  | CD11c | APC | 17-0116-42 | eBioscience |
|  | CD11c | PE | 12-0116-42 | eBioscience |
|  | CD123 | APC | 560087 | BD Biosciences |
|  | CD123 | PE | 554529 | BD Biosciences |
|  | CD123 | FITC | 558663 | BD Biosciences |
|  | CD14 | PB | 558121 | BD Biosciences |
|  | CD16 | AF700 | 557920 | BD Biosciences |
|  | CD16 | PO | MHCD1630 | Thermo Fisher |
|  | CD177 | FITC | MCA2045 | Bio-Rad AbD Serotec |
|  | CD19 | PE-Cy7 | IM3628U | Beckman Coulter |
|  | CD1c | PE | 130-090-507 | Miltenyi Biotec |
|  | CD20 | FITC | 555622 | BD Biosciences |
|  | CD3 | PE-Cy7 | 557749 | BD Biosciences |
|  | CD40 | APC-H7 | 561211 | BD Biosciences |
|  | CD45 | PerCP | 558411 | BD Biosciences |
|  | CD45 | PE-TR | MHCD0417 | Thermo Fisher |
|  | CD8 | APC-H7 | 348813 | BD Biosciences |
|  | CD8 | PE-Cy7 | 335787 | BD Biosciences |
|  | CD86 | AL700 | 561124 | BD Biosciences |
|  | HLADR | PO | MHLDR30 | Thermo Fisher |
|  | HLADR | ECD | IM3636 | Beckman Coulter |
|  | IFNg | FITC | 552887 | BD Biosciences |
|  | NKp46 | PE | A66902 | BD Biosciences |
